# Deep palaeoproteomic profiling of archaeological human brains

**DOI:** 10.1371/journal.pone.0324246

**Published:** 2025-05-28

**Authors:** Alexandra Morton-Hayward, Sarah Flannery, Iolanda Vendrell, Roman Fischer

**Affiliations:** 1 Department of Earth Sciences, University of Oxford, Oxford, United Kingdom; 2 Target Discovery Institute, Nuffield Department of Medicine, University of Oxford, Oxford, United Kingdom; Aarhus University, DENMARK

## Abstract

Palaeoproteomics leverages the persistence, diversity, and biological import of ancient proteins to explore the past, and answer fundamental questions about phylogeny, environment, diet, and disease. These insights are largely gleaned from hard tissues like bone and teeth, as well-established protocols exist for extracting ancient proteins from mineralised tissues. No such method, however, exists for the soft tissues, which are underexplored in palaeoproteomics given permission for destructive analysis routinely depends on a proven methodology. Considering less than one-tenth of all human proteins are expressed in bone, compared to three-quarters in the internal organs, the amount of biological information presently inaccessible is substantial. We address this omission with an optimised LC-FAIMS-MS/MS workflow yielding the largest, most diverse palaeoproteome yet described. Using archaeological human brains, we test ten protocols with varied chemistries and find that urea lysis effectively disrupts preserved membrane regions to expose low-abundant, intracellular analytes. Further, we show that ion mobility spectrometry improves unique protein identification by as much as 40%, and represents a means of “cleaning” dirty archaeological samples. Our methodology will be useful for improving protein recovery from a range of ancient tissues and depositional environments.

## Introduction

Proteins are long-lived biomolecules able to persist for millions of years in the archaeological record [1–3]. Their longevity derives in part from their structure, comprising long chains of 20 standard amino acids (each of which possesses a chemically distinct side chain) folded into complex secondary, tertiary and quaternary structures, which are compounded by an array of possible post-translational modifications (PTMs). This compact architecture packs the same amount of sequence information as DNA into approximately one-sixth the number of atoms and, with fewer chemical bonds, degrades more slowly than the double helix that encodes it [4]. Ancient protein sequences thus trace the same phylogenetic relationships as DNA, with the additional advantage that preservation of *in vivo* PTMs yields physiological information on the lived experience of long-dead lineages [5]. That is, where DNA provides the potential, proteins provide the record. Their persistence and biochemical diversity make proteins an ideal vehicle for navigating the recent and deep past [6], and the challenge of palaeoproteomics is to retrieve, identify, and make sense of their remnants as they transition from the biosphere to the lithosphere [4].

To be detected and analysed, proteins must first be extracted. Sample preparation remains the major bottleneck in any bottom-up proteomics workflow given the complexity of biological samples and the multi-stage processes associated with it [7–9], which typically include cell lysis, protein denaturation, disulfide bond reduction, sulfhydryl group alkylation, and digestion of proteins into peptides for efficient separation by liquid chromatography (LC) and detection by mass spectrometry (MS) [10]. This complexity is only exacerbated in an archaeological context, where the action of time has further depleted and degraded the ancient proteome [11]. Nonetheless, ancient proteomes have been retrieved from hard tissues like bone and teeth in diverse taxa [4], and a handful of studies have investigated the efficiency of different methods for extracting ancient proteins from these remains [12–14]. However, archaeological soft tissues remain underexplored as repositories of ancient proteins: those few to-date have been restricted largely to hair [15] and skin [16–18], and no method optimisation has been conducted beyond extraction from keratinised and collagenous tissues. Ancient hair, skin and bone proteomes largely comprise keratins and collagens, and less than 9% of all human proteins are expressed in bone (1,730/20,162) [19]. By contrast, 76% are expressed in the brain (15,331/20,162) [20], and the ancient brain proteome is thus an order of magnitude more diverse, reflective of its functional complexity [21]. The same is likely true of other internal organs preserved in the archaeological record, such as the intestines (75%) [22,23], stomach (72%) [24,25], kidneys (71%) [26,27], lungs (71%) [28,29] and heart (68%) [30,31].

More than 4,400 human brains have been excavated from an array of environments worldwide, some up to 12,000 years old [32]; just two, however, have been probed by LC-MS/MS [21,33]. Soft tissues of the internal organs (and particularly the brain) comprise substantially greater proportions of lipid-linked membrane proteins than hard tissues [20]. Anchored in the lipid bilayer environment, membrane proteins comprise one or more alpha-helical domains [34] whose hydrophobic nature can cause aggregation in aqueous solutions [35], which makes sample preparation challenging. Membrane proteins are often extracted and kept soluble with strong denaturants (e.g., urea) or ionic detergents (e.g., sodium dodecyl sulphate [SDS]) which, when maintained above their critical micelle concentration, effectively disrupt the lipid bilayer [36]. While such agents may facilitate extraction, they can inhibit proteolysis and interfere with separation by LC and detection by MS, hampering protein recovery [9,37]. As such, a range of sample clean-up methods exist at both the protein- and peptide-level (i.e., conducted before or after proteolysis) that aim to remove reagents that would otherwise negatively impact downstream analyses [38]. However, additional steps cause sample losses, which it is crucial to minimise in cases where low starting material availability must be a consideration [39], such as with archaeological tissues. Moreover, choices made at each stage of the proteomics workflow affect the composition of the recovered proteome: for example, reagents can selectively enrich or deplete functional classes of proteins and cellular components, depending on the underlying chemistry [40–42].

**Fig 1:**
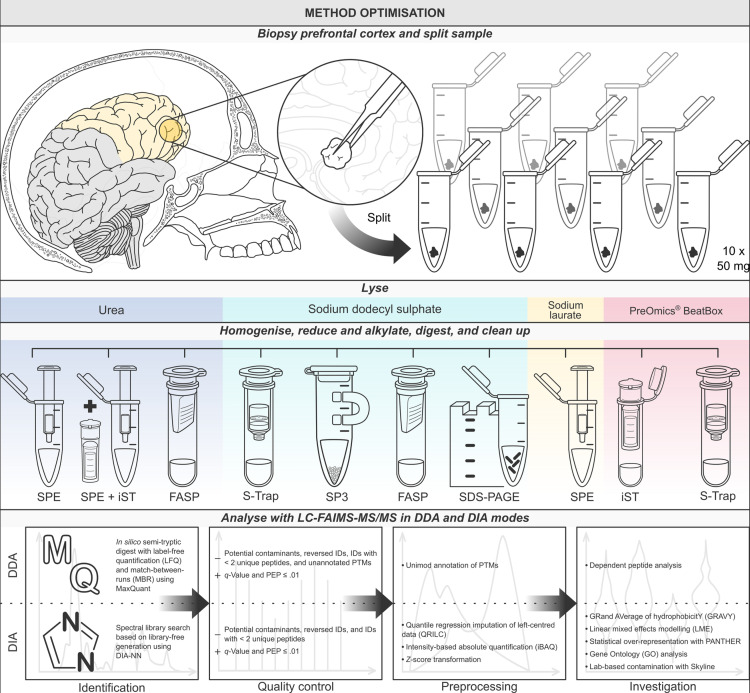
Overview of the study design. A single cortical biopsy was taken from the anterior right frontal lobe and separated into ten 50 mg samples, which were lysed with four different extraction methods (urea, SDS, SL and PreOmics® BeatBox) before homogenisation by bead-beating. After reduction, alkylation, and digestion with trypsin, samples were processed with one of eight clean-up methods either in solution (iST, SPE, SPE + iST), in-gel (SDS-PAGE), on-filter (FASP, S-Trap) or on-bead (SP3). Samples were analysed by LC-FAIMS-MS/MS in DDA and DIA modes and, based on preliminary testing with a range of software (see Table S1 in S1 File), the final DDA dataset was processed in MaxQuant [49], and the DIA dataset in DIA-NN [47].

We test ten protocols for ancient protein recovery from archaeological brain tissues using a bottom-up LC-MS/MS approach, comparing the effectiveness of a range of extraction buffers and clean-up strategies (Fig 1). The former include urea, SDS and sodium laurate (SL); while the latter include the in-StageTip method (iST) [43], solid-phase extraction (SPE), suspension trapping (S-Trap) [44], filter-aided sample preparation (FASP) [45], single-pot solid-phase-enhanced sample preparation (SP3) [46], and in-gel purification with SDS-polyacrylamide gel electrophoresis (SDS-PAGE). We assess the use of a high-field asymmetric-waveform ion mobility spectrometry (FAIMS) source at different compensation voltages to reduce chemical noise and improve detection of low-abundant proteins. Finally, the range of laboratory-based options available to the protein chemist is paralleled by the array of analytical software available to the bioinformatician: we test protein identification and relative quantification with DIA-NN [47], FragPipe [48], MaxQuant [49] and PEAKS® Studio (Bioinformatics Solutions, Inc.). We assess the efficacy, efficiency (i.e., performance relative to ease of use), and suitability of all options for broad application in palaeoproteomics, comparing protocols on the basis of numbers of identified precursors, peptides and proteins, intensity-based absolute quantification (iBAQ), protein scoring, hydropathy and isoelectric point, levels of reagent-based contamination, and functional and cellular enrichment biases.

## Materials and methods

### Archaeological brain material

Approximately 4,500 individuals were excavated from the site of the former Blackberry Hill Hospital in the Fishponds area of Bristol, UK (2018–2022, Cotswold Archaeology), one in ten of which evidenced preserved brain tissues (*n* = 456). Originally built as Stapleton Prison (1779–1814) to house prisoners of war from Britain’s conflicts with France, Spain, the Netherlands and the USA throughout the late 18th century, the site became a hospital during Bristol’s 1832 cholera outbreak, and was converted to the Stapleton Workhouse shortly thereafter (1837–1890). In the 20th century, it evolved into Manor Park Hospital, later Blackberry Hill Hospital, before its closure in 2007.

Immediately post-excavation, brains were refrigerated at 4 °C in airtight containers, without washing or preservative treatment, and acquisitioned by the Duckworth Laboratory (University of Cambridge). One brain (SK 17999) was selected for investigation based on the exceptional quality of its preservation, possessing identifiable cerebral hemispheres with intact sulci and gyri that facilitated confident and reproducible sampling. A single cortical biopsy was taken from the anterior right frontal lobe, which was separated into ten 50 mg samples; these were stored at –80 °C prior to analysis. Permission for destructive sampling was sought and obtained from the Duckworth Laboratory, and ethical approval from the Central University Research Ethics Committee at the University of Oxford (R80617/RE001). All necessary permits were obtained for the described study, which complied with all relevant regulations.

### In-solution

#### PreOmics® BeatBox iST.

The sample was processed according to the iST Sample Preparation Kit protocol (v.4.0; PreOmics®) [50]. A 50 mg aliquot of brain was lysed with 50 mg glass beads in 100 µL of LYSE, using BeatBox sonication for 10 cycles of 30 sec. Proteins were further denatured at 95 °C and 1,000 rpm for 10 min in an Eppendorf ThermoMixer® C (Thermo Fisher Scientific). Digestion was performed with 50 µL DIGEST at 37 °C and 500 rpm for 3 hr. The sample was acidified with 100 µL STOP then clarified at 20 °C and 500 rpm for 1 min, followed by centrifugation at 16,000 rcf for 1 min. Peptides were purified using the PreOmics® iST method. Eluted peptides were dried by vacuum centrifugation for storage at –20 °C, then reconstituted in 50 µL LC-LOAD at 20°C and 500 rpm for 5 min prior to LC-MS analysis.

#### Urea with SPE (and iST).

A 50 mg aliquot of brain was lysed in 8 M urea, 100 mM triethylammonium bicarbonate (TEAB) pH 8.5, using a Precellys® 24 Touch homogeniser (Bertin Technologies) for three cycles of 30 sec at 5,500 rpm. The sample was placed on ice between cycles to reduce heating. Proteins were further denatured at 20°C and 1,000 rpm for 30 min in an Eppendorf ThermoMixer® C (Thermo Fisher Scientific). Cysteine reduction and alkylation were performed at 20 °C and 1,000 rpm for 30 min with 10 mM tris(2-carboxyethyl)phosphine (TCEP) and 50 mM iodoacetamide (IAA), respectively. The urea concentration was diluted to 1.5 M with 50 mM TEAB pH 8.5. Digestion was performed with 1 µg trypsin (MS-grade, Promega) overnight at 37 °C and 1,250 rpm. The sample was acidified with TFA to 1% then clarified by centrifugation at 22,000 rcf for 10 min. Peptides were diluted 10-fold in 0.1% trifluoroacetic acid (TFA), 2% acetonitrile (ACN) then purified by SPE (SOLA^TM^ SPE, Thermo Fisher Scientific). The sample was split: subsample A underwent no further clean-up, and eluted peptides were dried by vacuum centrifugation for storage at –20 °C, then reconstituted in 0.1% formic acid (FA), 3% ACN prior to LC-MS analysis. Subsample B was subjected to the PreOmics® iST method as described in the iST Sample Preparation Kit protocol (v.4.0; PreOmics®) [50], and eluted peptides dried by vacuum centrifugation for storage at –20 °C, then reconstituted in 50 µL LC-LOAD at 20 °C and 500 rpm for 5 min prior to LC-MS analysis.

#### Sodium laurate with SPE.

A 50 mg aliquot of brain was lysed in 1% sodium laurate (SL), 100 mM TEAB pH 8.5, using a Precellys® 24 Touch homogeniser (Bertin Technologies) for three cycles of 30 sec at 5,500 rpm. The sample was placed on ice between cycles to reduce heating. Proteins were further denatured in an ultrasonic bath at 4 °C for 10 min. Cysteine reduction and alkylation were performed at 20 °C without shaking for 30 min with 10 mM TCEP and 20 mM IAA, respectively. Digestion was performed with 1 µg trypsin (MS-grade, Promega) at 37 °C and 1,000 rpm for 3 hr. The sample was acidified with TFA to 1% then clarified by centrifugation at 22,000 rcf for 5 min. To remove SL, a volume of ethyl acetate equal to the volume of supernatant was added and the sample centrifuged at 15,700 rcf for 5 min, before removal of the lower phase and repetition of this step. Peptides were diluted 10-fold in 0.1% TFA, 2% ACN then purified by SPE (SOLA^TM^ SPE, Thermo Fisher Scientific). Eluted peptides were dried by vacuum centrifugation for storage at –20 °C, then reconstituted in 0.1% FA, 3% ACN prior to LC-MS analysis.

### In-gel

#### SDS-PAGE.

A 50 mg aliquot of brain was lysed in 5% SDS, 100 mM TEAB pH 8.5, using a Precellys® 24 Touch homogeniser (Bertin Technologies) for three cycles of 30 sec at 5,500 rpm. The sample was placed on ice between cycles to reduce heating. NuPAGE^TM^ LDS (4x) sample buffer (Invitrogen^TM^, Thermo Fisher Scientific) was added with 200 mM dithiothreitol (DTT) to a final concentration of 1%, before incubating at 20 °C and 2,000 rpm for 1 hr. The sample was loaded into six lanes of a 1 mm NuPAGE^TM^ Bis-Tris gel, mounted in a Mini Gel Tank and loaded with NuPAGE^TM^ MES SDS (20x) running buffer (all Invitrogen^TM^, Thermo Fisher Scientific). The gel was run at 120 V for 10 min before removal and staining with InstantBlue^TM^ dye (Sigma-Aldrich). Each gel lane was excised into approximately 1 mm^2^ cubes and combined, before submersion in 5% acetic acid at 20 °C and 1,000 rpm overnight in an Eppendorf ThermoMixer® C (Thermo Fisher Scientific). Cysteine reduction was performed with 10 mM DTT at 20 °C without shaking for 30 min, followed by alkylation with 50 mM IAA at 20 °C without shaking for 30 min. The gel pieces were dehydrated with ACN at 20 °C and rehydrated with 100 mM ammonium bicarbonate (ambic) at 20 °C prior to digestion, which was performed with 1 µg trypsin (MS-grade, Promega) in 50 mM ambic overnight at 37 °C without shaking. Peptides were eluted with 5% FA, 50–85% ACN, dried by vacuum centrifugation for storage at –20 °C, then reconstituted in 0.1% FA, 3% ACN prior to LC-MS analysis.

### On-filter

#### PreOmics® BeatBox with S-Trap^TM^ micro.

A 50 mg aliquot of brain was lysed in 5% SDS reducing buffer, 10 mM TCEP and 100 mM TEAB pH 8.5, using BeatBox sonication (PreOmics®) for 10 cycles of 30 sec. Cysteine alkylation was performed with 20 mM IAA at 20 °C without shaking for 30 min. The sample was acidified with phosphoric acid to 1.2% concentration, and loaded onto an S-Trap^TM^ micro (Protifi, Biosys Technologies, Inc.). Digestion was performed on-filter in 50 mM TEAB pH 8.5 with 1 µg trypsin (MS-grade, Promega) overnight at 37 °C without shaking. The sample was eluted sequentially with 50 mM TEAB pH 8.5, 0.2% FA, then 0.2% FA, 50% ACN. Eluted peptides were dried by vacuum centrifugation for storage at –20 °C, then reconstituted in 0.1% FA, 3% ACN prior to LC-MS analysis.

#### SDS with S-Trap^TM^ micro.

A 50 mg aliquot of brain was lysed in 5% SDS reducing buffer, 10 mM TCEP and 100 mM TEAB pH 8.5, using a Precellys® 24 Touch homogeniser (Bertin Technologies) for three cycles of 30 sec at 5,500 rpm. The sample was placed on ice between cycles to reduce heating. Cysteine alkylation was performed with 20 mM IAA at 20 °C without shaking for 30 min. The sample was acidified with phosphoric acid to 1.2% concentration, and loaded onto an S-Trap^TM^ micro (Protifi, Biosys Technologies, Inc.). Digestion was performed on-filter in 50 mM TEAB pH 8.5 with 1 µg trypsin (MS-grade, Promega) overnight at 37 °C without shaking. The sample was eluted sequentially with 50 mM TEAB pH 8.5, 0.2% FA, then 0.2% FA, 50% ACN. Eluted peptides were dried by vacuum centrifugation for storage at –20 °C, then reconstituted in 0.1% FA, 3% ACN prior to LC-MS analysis.

#### Urea with FASP.

A 50 mg aliquot of brain was lysed in 8 M urea, 100 mM TEAB pH 8.5, using a Precellys® 24 Touch homogeniser (Bertin Technologies) for three cycles of 30 sec at 5,500 rpm. The sample was placed on ice between cycles to reduce heating. The sample was filtered with a Sartorius Vivacon^TM^ 500 30 kDa molecular-weight cut-off (MWCO) filter (Thermo Fisher Scientific), centrifuged at 14,300 rcf for 10 min. Cysteine reduction and alkylation were performed with 10 mM TCEP and 50 mM IAA respectively, on-filter in 8 M urea, 100 mM TEAB pH 8.5 at 20 °C without shaking for 30 min. The filter was washed three times with 50 mM TEAB pH 8.5, and digestion performed with 1 µg trypsin (MS-grade, Promega) overnight at 37 °C without shaking. Peptides were eluted with three cycles of centrifugation at 14,300 rcf for 10 min, with dilution in 0.1% TFA, 50% ACN. Eluted peptides were dried by vacuum centrifugation for storage at –20 °C, then reconstituted in 0.1% FA, 3% ACN prior to LC-MS analysis.

#### SDS with FASP.

A 50 mg aliquot of brain was lysed in 5% SDS, 100 mM TEAB pH 8.5, using a Precellys® 24 Touch homogeniser (Bertin Technologies) for three cycles of 30 sec at 5,500 rpm. The sample was placed on ice between cycles to reduce heating. The sample was filtered with a Sartorius Vivacon^TM^ 500 30 kDa MWCO filter (Thermo Fisher Scientific), centrifuged at 14,300 rcf for 10 min. Cysteine reduction and alkylation were performed with 10 mM TCEP and 50 mM IAA respectively, on-filter in 8M urea, 100 mM TEAB pH 8.5 at 20 °C without shaking for 30 min. The filter was washed three times with 50 mM TEAB pH 8.5, and digestion performed with 1 µg trypsin (MS-grade, Promega) overnight at 37 °C without shaking. Peptides were eluted with three cycles of centrifugation at 14,300 rcf for 10 min, with dilution in 0.1% TFA, 50% ACN. Eluted peptides were dried by vacuum centrifugation for storage at –20 °C, then reconstituted in 0.1% FA, 3% ACN prior to LC-MS analysis.

### On-bead

#### SP3.

A 50 mg aliquot of brain was lysed in 0.5% SDS using a Precellys® 24 Touch homogeniser (Bertin Technologies) for three cycles of 30 sec at 5,500 rpm. The sample was placed on ice between cycles to reduce heating. Cysteine reduction was performed with 5 mM DTT at 20 °C without shaking for 30 min, followed by alkylation with 20 mM IAA at 20 °C without shaking for 30 min. The sample was combined with 3 μL of SP3 beads (1:1 hydrophobic:hydrophilic) and ACN added to a final concentration of 70%, before mixing at 20 °C and 1,000 rpm for 18 min. The sample was placed on a magnetic rack and the beads allowed to settle for 2 min, before being washed three times with 100% ACN. Beads were resuspended in 50 mM TEAB pH 8.5 and digestion performed with 1 µg trypsin (MS-grade, Promega) overnight at 37 °C and 1,000 rpm. The sample was acidified with TFA to 1% and eluted peptides were dried by vacuum centrifugation for storage at –20 °C, then reconstituted in 0.1% FA, 3% ACN prior to LC-MS analysis.

### Data acquisition

LC-FAIMS-MS/MS was performed on a Vanquish Neo UHPLC coupled to an Orbitrap^TM^ Ascend Tribrid^TM^ mass spectrometer equipped with a FAIMS Pro Duo interface (all Thermo Fisher Scientific). This system was benchmarked against a Hela cell lysate to ensure optimal performance. The Vanquish Neo was operated in “Trap and Elute” mode, using a PepMap^TM^ Neo Trap Cartridge (5 mm x 300 μm) and EASY-Spray^TM^ PepMap^TM^ Neo UHPLC Column (50 cm x 75 μm, 1500 bar; both Thermo Fisher Scientific). Tryptic peptides were trapped and separated with a 60 min linear gradient; from 2% to 18% buffer B (0.1% FA in ACN) in buffer A (0.1% FA in H_2_O) over 40 min, from 18% to 35% B over 20 min, and from 35% to 99% B over 15 min at 300 nL/min flow rate.

In data dependent acquisition (DDA) mode, MS1 spectra were acquired in the Orbitrap^TM^ at 120K resolution between 380 and 1500 *m/z*, with an automatic gain control (AGC) target of 4e5 ions, a maximum injection time of 251 ms, radio frequency (RF) lens array at 30%, and advanced peak determination toggled on. Precursor ions were fragmented using high energy collisional dissociation with 30% normalised collision energy (NCE). MS2 spectra were acquired in the ion trap with rapid scan mode, a quad isolation window of 1.2 *m/z*, an AGC target of 5e3 ions, and a maximum injection time of 50 ms. The FAIMS Pro Duo was operated at standard resolution with a carrier gas flow rate of 3.8 L/min and cycling through two compensation voltages (CV, –40 and –55 V), with one sec per CV. In data independent acquisition (DIA) mode, MS1 spectra were acquired in the Orbitrap^TM^ at 45K resolution between 350 and 1650 *m/z*, with an AGC target of 5e5 ions, a maximum injection time of 91 ms, and RF lens array at 30%. MS2 spectra were acquired using the tMSn scan function at 30K resolution over 40 scan windows (with variable isolation width) covering the full 350–1650 *m/z* range, with an AGC target of 4e6 ions, an automatic maximum injection time, and 30% NCE. The FAIMS source was operated at standard resolution with a carrier gas flow rate of 3.8 L/min, and compensation voltage of –45 V.

### Data analysis

Eight searches of all LC-FAIMS-MS/MS raw data files acquired in DDA (*n* = 10) and DIA mode (*n = *10) were conducted using a range of software packages for protein identification and quantification (see, Table S1 in S1 File). In all cases, data were searched against the human brain proteome with semi-specific tryptic digestion and up to three missed cleavages allowed [51], with the following modifications: carbamidomethylation of Cys (fixed), N-terminal acetylation (variable), oxidation of Met and Pro (variable), and deamidation of Asn, Gln and Arg (variable). The human brain proteome was retrieved by downloading all protein-coding genes detected in the human brain (*n* = 15,331) from the Human Protein Atlas (v.23.0, accessed 15.02.2024) [20], and converting Ensembl identifiers to UniProt accession numbers using the UniProt ID mapping tool (v. 2024.01) [52]. Having removed duplicates, the remaining proteins (*n* = 15,049) were downloaded as a FASTA file.

A search of DDA data based on *in silico* FASTA digest with label-free quantification and match-between-runs in MaxQuant (v.2.4.3.0) [49] yielded the fewest unique proteins identified at 1% FDR but the most information in terms of protein modification, and all further analyses of DDA data were performed on this dataset using the R programming language (v.2024.04.2). Global peptide and protein lists were removed of potential contaminants, reversed identifications, and identifications with < 2 unique peptides, then filtered for *q*-value and posterior error probability (PEP) ≤.01. Modified specific and dependent peptide lists were removed of unannotated modifications and those corresponding to < 2 unique peptides, then filtered for *q*-value and PEP ≤ .05.

Spectral library search of DIA data based on library-free generation with DIA-NN (v.1.9) [47] yielded the most confident identifications at 1% FDR (mean global *Q*-value: 2.7 x 10^-3^), and all further analyses of DIA data were conducted on this dataset. DIA data were preprocessed using the R package *DIAgui* (v.1.4.2) [53], which implements the *iq* package (v.1.9.12) [54] for calculating proteome-wide, label-free quantification by delayed normalisation and maximal peptide ratio extraction (*aka*. MaxLFQ) [55], and the *imputeLCMD* package for left-centred missing data imputation [56]. Precursors were filtered using the following parameters: precursor *q*-value ≤ .01, protein group *q*-value ≤ .01, and protein and gene-group *q-*values ≤ 1. Peptides and protein groups were filtered with the same parameters. iBAQ was performed on protein groups [57]: data were log_2_-transformed to normalise the data range, and missing data imputed using quantile regression (QRILC) [58]. A *z*-score transformation on samples was subsequently performed to enable comparison of proteomic profiles across protocols: this approach standardises the data for each protocol by giving each sample a mean of 0 and a standard deviation (SD) of 1 across all proteins [59].

GRand AVerage of hydropathY (GRAVY) scores were calculated using the Kyte-Doolittle hydrophobicity scale [60], and the average isoelectric point (pI) using Isoelectric Point Calculator (IPC) 2.0 [61]. Both measures were calculated at the protein-level for the subset of proteins identified in all protocols (*n* = 98), and at the peptide-level for peptides corresponding to these proteins recovered by each protocol. This “common proteome” allowed for a comparison in terms of the relative hydropathy of recovered peptides corresponding to these proteins against a shared pool. Statistical testing was performed to probe significant differences between methods: the data failed a Shapiro-Wilk test for normality (*W*[10] =.984, *p* = 2.5 x 10^-2^) and Levene’s test for homogeneity of variance (*F*[10] = 6.93, *p* = 7.6 x 10^-11^). Given the data is not independent (all protocols having been conducted on the same biopsy), and given the inequality of proteome size across protocols, a linear mixed-effects model with Satterthwaite *t-*tests (*n* = 10) and *post-hoc* pairwise comparison using Bonferroni correction at 95% confidence interval (CI) was developed for both GRAVY and pI scores, using the R packages *lme4* (v.1.1.35.5) [62] and *emmeans* (v.1.10.3) [63]. It should be noted that any statistically significant differences must be treated with caution given that, due to the irreplaceable nature of archaeological material, we were unable to perform replicate extractions (see Limitations).

Over- and under-representation (i.e., the difference between the number of observed *vs.* expected proteins) was investigated at the protein class level using a Fisher’s exact test and Bonferroni correction with the Protein ANalysis THrough Evolutionary Relationships (PANTHER) classification system (v.19.0) [64]. Gene Ontology (GO) functional enrichment analysis was also performed in PANTHER using a Fisher’s exact test and Bonferroni correction, to investigate enrichment in cellular components [65]. Finally, levels of common laboratory contamination were assessed using Skyline (v.24.1) [66] to extract features from raw LC-FAIMS-MS/MS data corresponding to potential targets in the Molecular Contaminant List [67], and extracted ion chromatograms processed using the *HowDirty* R package (v.0.2.1) [68].

## Results

### Benchmarking protocol performance with ion mobility spectrometry

**Fig 2:**
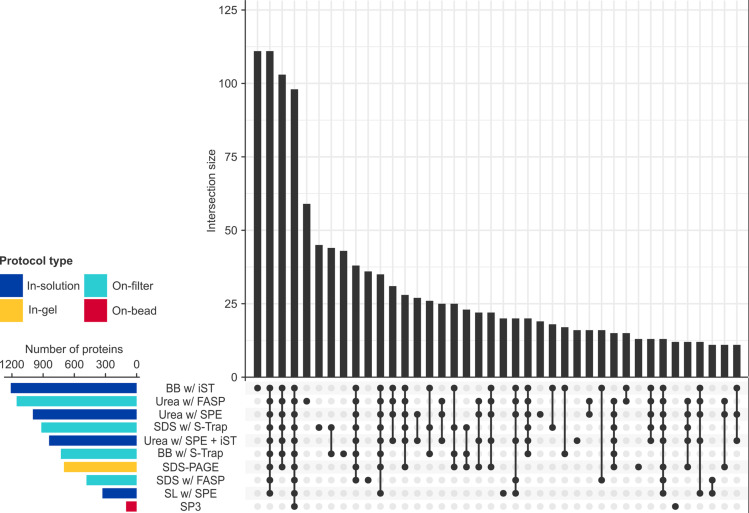
Total numbers of proteins retrieved with each protocol. Upset plot of the number of unique proteins at 1% FDR and *q*-value ≤ .01 recovered by each protocol (*bottom left*), and the number (*top*) and nature (*bottom right*) of intersections in the data.

While the number of unique proteins varied with the search strategy used (Fig S1 in S1 File), employing a FAIMS source increased protein detection by an average of 39.0% for DIA and 26.4% for DDA regardless of the search strategy, with a compensation voltage of –45 V yielding the most identifications (510 *vs.* –40 V: 487; Fig S2 in S1 File). Total numbers of proteotypic precursors, peptides and proteins recovered with each protocol in DIA are summarised in the Supplementary (Table S2 in S1 File), and the total numbers of unique proteins and overlap in proteomic profiles illustrated in Fig 2. The best-performing protocols in terms of protein recovery were the PreOmics® BeatBox iST (1,205) followed by urea with FASP (1,157), which also yielded the highest numbers of proteins recovered exclusively by one method (111 and 59 respectively). To the best of our knowledge, these represent the largest and most diverse palaeoproteomes identified in any archaeological material investigated to-date. SP3 yielded the fewest total proteins (104), with less than a third as many identifications as SL with SPE (339). Given the overall effectiveness of urea-based methods, we latterly tested SP3 following urea extraction, but this did not improve protein recovery appreciably (139). While more than half of identifications (53.7%, 797/1,483) were common to the best-performing methods, less than one-quarter (23.0%, 121/525) were shared by the worst-performing. Statistical over-representation testing for protein class of this common subset (Table S3 in S1 File) indicated statistically significant enrichment by almost 30x of tubulins (fold enrichment [FE]: 26.68, *p* = .03) and intermediate filaments (24.19, *p* = .003). GO functional enrichment analysis suggested that the majority of protocols (80.0%, 8/10) enriched first and foremost for cytoskeletal fibres such as these (Data S1 in S1 Dataset); however, urea-based protocols enriched principally for the mitochondrial ATP synthase complex (e.g., with SPE: 7.11, *p* = .008; with FASP: 6.58, *p* = .01).

**Fig 3:**
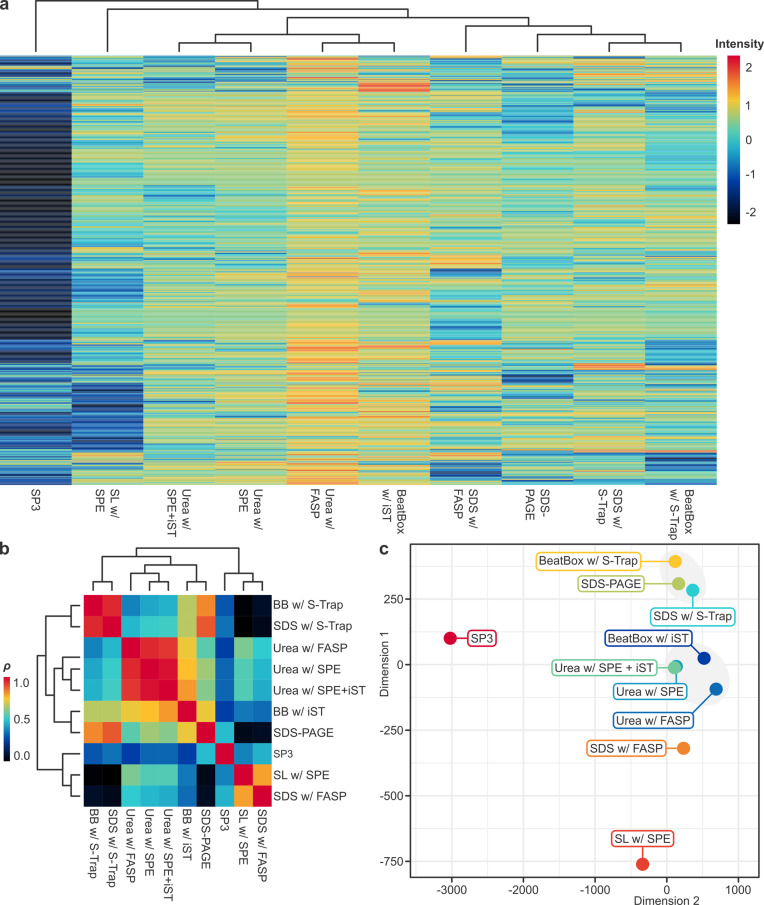
Unsupervised data clustering and multivariate ordination analyses. **(a)** Heat map with hierarchically clustered dendrograms of iBAQ by protocol. iBAQ data were log_2_-transformed and missing data imputed using QRILC, before a *z*-score transformation on samples was applied. Each row represents a recovered protein (*n* = 2,087) clustered by Manhattan distance with complete linkage (dendrograms not shown), and each column a protocol clustered by Euclidean distance. **(b)** Pearson’s correlation coefficient (*ρ*) matrix with hierarchically clustered dendrograms of iBAQ by protocol. As a normalised measure of covariance, *ρ* has a value between –1 (perfect negative correlation) and 1 (perfect positive correlation); however, note the restricted range illustrated (0.0–1.0), reflecting the fact that only positive correlations exist between protocols in our dataset. **(c)** Metric multidimensional scaling plot with Manhattan distance of iBAQ data by protocol. The distance between data points in two-dimensional Cartesian space correlates with the dissimilarity between those points. Grey circles delineate clusters.

Unsupervised hierarchical clustering of iBAQ by protocol (Fig 3a) illustrated the range of protein recovery; particularly, the extent of missing data with SP3, for which 94.5% (1,973/1,087) of proteins had a *z*-score < 0 (i.e., below average for the dataset), compared with the highest intensities yielded by urea with FASP, for which almost the same proportion (93.2%, 1,945/2,087) had a *z*-score > 0 (i.e., above average for the dataset). Correlation analysis with hierarchical clustering (Fig 3b) revealed only positive correlations (i.e., *ρ* > 0) between protocols, suggesting the identification and quantification of overlapping proteomic data, albeit with varying degrees of similarity. While direct reproducibility assessments were not performed, the observed correlations suggested that protocols sharing an extraction buffer or clean-up method yield comparable iBAQ intensities, preserving protein relative abundance proportions. For instance, the strongest correlations were observed between urea extraction on-filter and in-solution (FASP with SPE: *ρ* = .796, *p* < .001; FASP with SPE + iST: *ρ* = .778, *p* < .001), followed by clean-up with S-Trap, regardless of extraction buffer (*ρ* = 0.744, *p* < .001). In contrast, weak correlation between divergent protocols (e.g., SL with SPE *vs.* BeatBox with S-Trap: *ρ* = .037, *p* < .005) suggested disparities in quantification, potentially due to differences in protein denaturation, solubility, digestion or purification. Multidimensional scaling (Fig 3c) corroborated these trends with the emergence of two clusters. The first pertains largely to what might be considered “high-performance” protocols (≳ 1,000 protein identifications, exceeding the upper quartile for the dataset [984 proteins]), which share either urea extraction or iST clean-up; while the second pertains largely to “mid-performance” protocols (500–1,000 identifications, encompassing the median for the dataset [790]), which share SDS extraction. “Low-performance” protocols (≲ 500 identifications, below the lower quartile for the dataset [530]) appear as outliers in Cartesian space.

**Fig 4:**
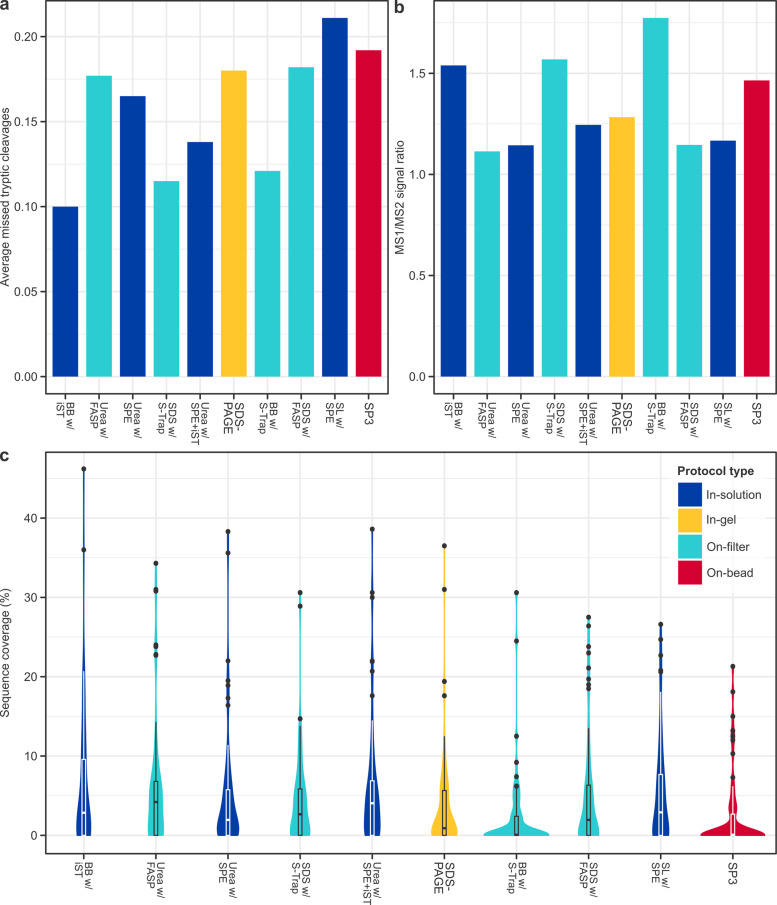
Measures of extraction efficiency. Bar charts of the **(a)** average missed tryptic cleavages and **(b)** MS1/MS2 signal ratios by protocol, gauges of digestion and fragmentation, respectively. **(c)** Violin plots of the range of sequence coverage of unique proteins identified at 1% FDR and *q*-value ≤ .01, retrieved by each protocol. Boxplots indicate the median and upper and lower quartiles, and black dots represent statistical outliers. Colours are consistent across panels, and in each panel protocols are ordered by, from left to right, highest to lowest total proteins.

Average missed tryptic cleavages and MS1/MS2 signal ratios may be tentatively used as gauges of digestion and fragmentation efficiency respectively (Fig 4a and 4b). On-filter clean-up methods with S-Trap evidenced among the lowest average missed cleavages (e.g., with SDS: 0.12) but the highest MS1/MS2 signal ratios (with SDS: 1.57), which suggest that, while proteolysis was relatively complete, fragmentation was not. The reverse pattern was observed for on-filter clean-up methods with FASP, which featured among the highest missed cleavages (e.g., with SDS: 0.18) and the lowest MS1/MS2 signal ratios (with SDS: 1.15). Mean sequence coverage across all protocols was relatively low (< 5%; Fig 4c), as expected for ancient proteins. The highest median coverage was obtained by extraction using urea (with FASP: 4.20%; with SPE + iST: 4.05%). BeatBox with S-Trap, although a mid-performance protocol in terms of protein recovery (735 identifications), yielded the lowest median (0.00%) and mean (2.22%) coverage, alongside SP3 (median: 0.00%; mean: 2.27%).

### Identifying biases in modification, hydropathy, isoelectric point and contamination

During diagenesis (the period after death and burial), ancient proteins are progressively degraded by hydrolysis and modified by deamidation (so-called, “diagenetic modifications”). DDA data was analysed to assess the extent of tryptic *vs.* non-tryptic peptide identification (i.e., the extent of diagenetic peptide bond cleavage), and the nature and extent of protein modification. Approximately two-thirds of the dataset comprised tryptic peptides, being cleaved C-terminal to either Lys (33.4%, 194/580) or Arg (32.8%, 190/580); of the remaining non-tryptic third, the most common cleavage sites were C-terminal to aliphatic Leu (5.2%, 30/580) and aromatic Phe (4.5%, 26/580). More than half of all non-tryptic peptides (53.6%, 105/196) corresponded to myelin proteolipid protein (Fig S3 in S1 File), with the myelin protein class more than 40-fold enriched (FE: 43.85, *p* = 7.7 x 10^-3^). SDS with S-Trap and urea with FASP yielded the highest numbers of non-tryptic peptides (73 and 67 respectively), while BeatBox with iST yielded the fewest (29) after SP3 (21), despite being the best-performing protocol in terms of protein recovery.

**Fig 5:**
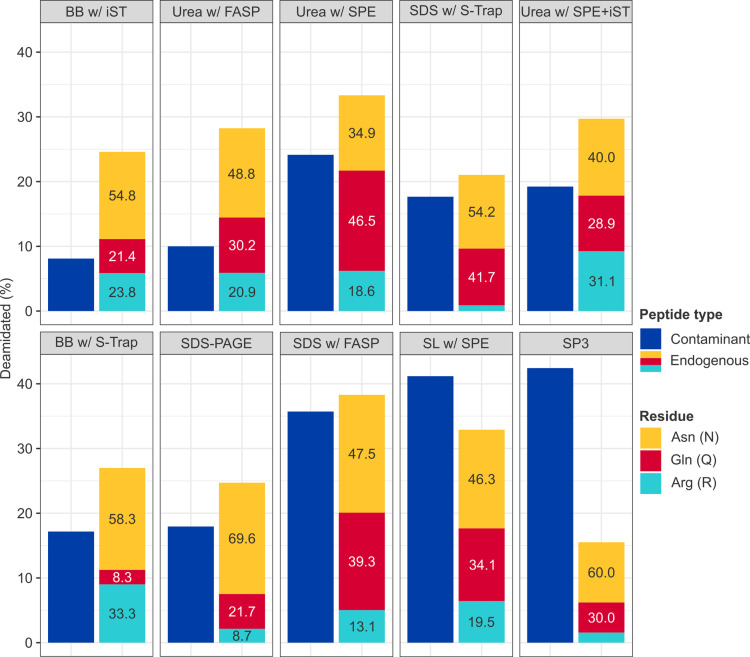
Nature and extent of deamidation. Percentage of deamidated contaminant (*left*) and endogenous (*right*) peptides at 1% FDR and *q*-value ≤ .05 by protocol (from left to right, highest to lowest total proteins). Endogenous peptides are coloured by the affected amino acid, with numbers indicating the percentage each type of deamidation represents of the total.

**Fig 6:**
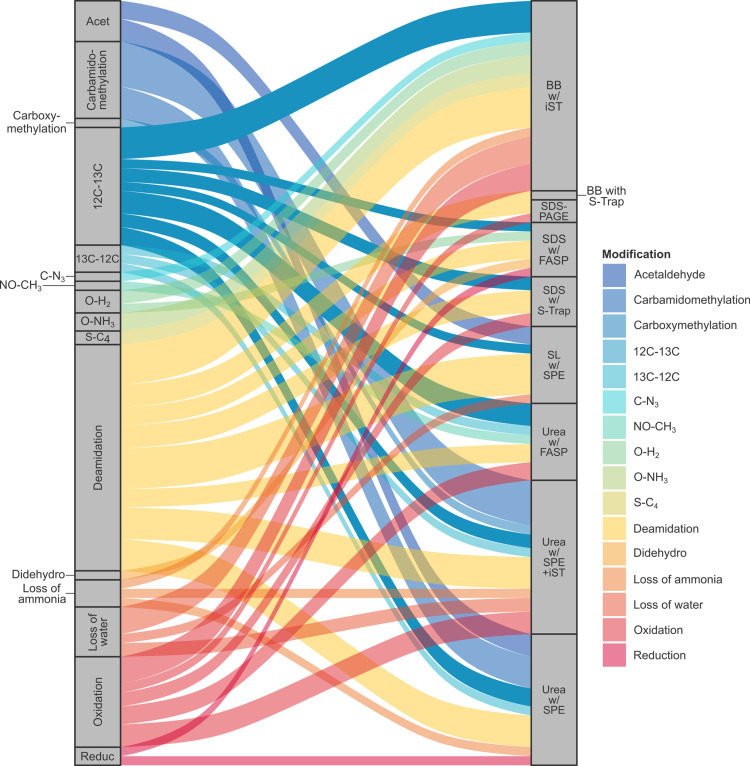
Discovery of covalent modifications. Alluvial diagram of modifications (*left*) to ≥ 2 unique peptides per protocol (*right*), identified by dependent peptide search. SP3 is not shown, since no identifications met the ≥ 2 unique peptides threshold.

The nature and extent of deamidation – a common diagenetic modification to ancient proteins, but also potentially induced during sample preparation – was assessed for both endogenous and contaminant peptides (Fig 5). In contrast to high- and mid-performance protocols, low-performance protocols evidenced greater proportions of deamidated contaminants than endogenous peptides (e.g., SP3: 42.4% *vs.* 15.5%). On average, approximately half of deamidated sites in endogenous peptides affected Asn (51.4%), approximately one-third Gln (30.2%), and approximately one-fifth Arg (18.3%). Functional enrichment analysis suggested that deamidation of Arg disproportionately affected cellular components associated with the myelin sheath (strength: 2.0; *p* = 4.5 x 10^-6^). In addition to modifications explicitly searched for, a dependent peptide search identified > 200 annotated modifications to ≥ 2 peptides (Fig 6 and Table S4 in S1 File), of which the most frequent were loss of water (*n* = 14), acetaldehyde adduct formation (*n* = 11), and metal-catalysed oxidation (*n* = 8).

**Fig 7:**
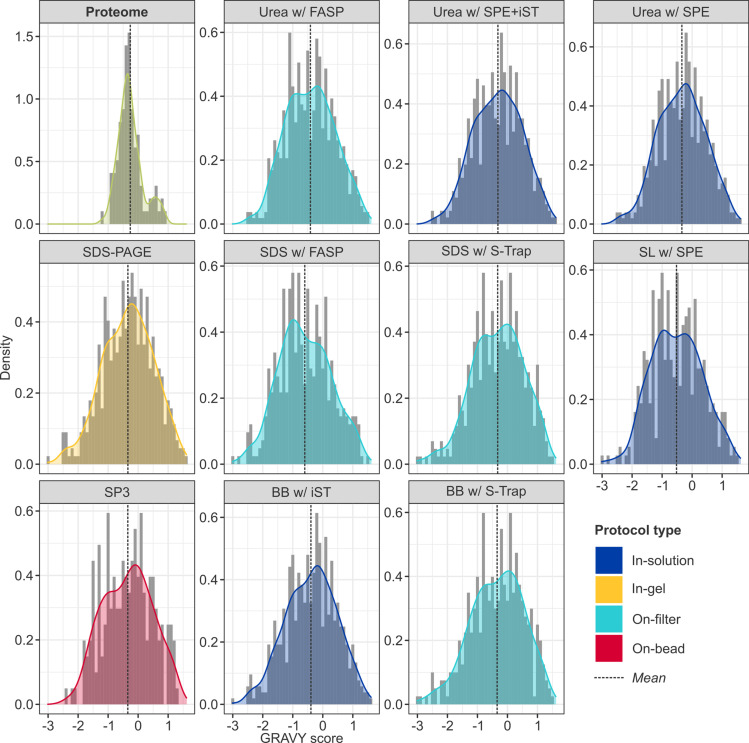
Distribution of GRAVY scores by protocol. Distribution of GRAVY scores calculated at the protein-level for proteins retrieved in all protocols (the “common proteome”, *green*), and at the peptide-level for proteins recovered by each protocol, with smoothed density curves. Given the variability in sample sizes, data has been normalised to reflect relative frequency rather than absolute counts (note the differing *y*-axes) and facilitate visual pairwise comparison of extraction buffers (broadly, horizontal rows; e.g., urea, *top*) and clean-up strategies (broadly, vertical columns; e.g., SPE, *right*). Dashed lines reflect the mean GRAVY score: a higher score indicates more hydrophobic peptides, and a lower score more hydrophilic peptides.

**Fig 8:**
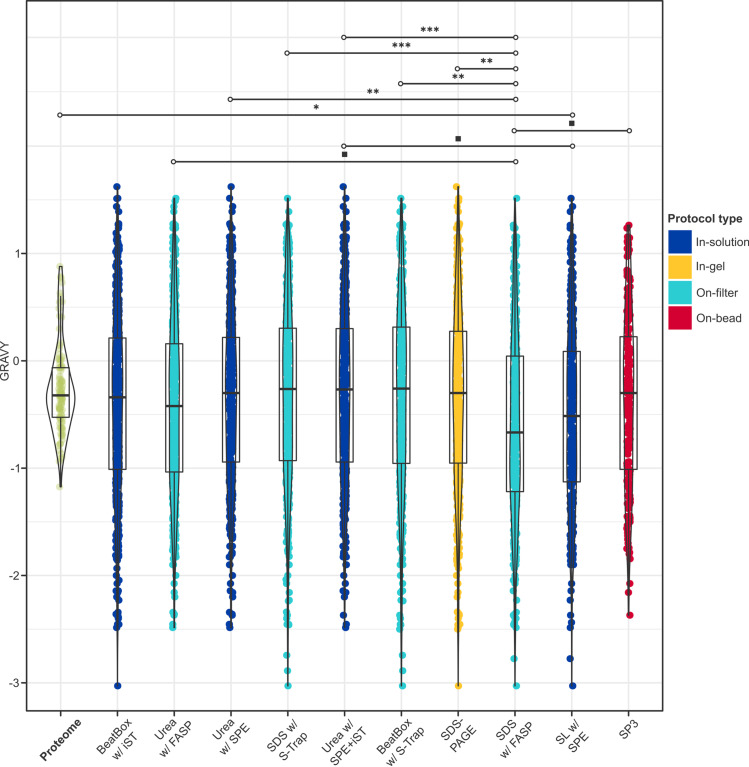
Hydropathic profiles retrieved with each protocol. Results of a linear mixed effects model with Satterthwaite *t*-tests (*n*_obs_ = 4,383) and *post-hoc* pairwise comparisons (*n* = 55) with Bonferroni correction at 95% CI with respect to GRAVY score, calculated at the protein-level for proteins retrieved in all protocols (the “common proteome”, *green*), and at the peptide-level for proteins recovered by each protocol (from left to right, highest to lowest total proteins). Only statistically significant comparisons are shown (for all other comparisons, see Table S6 in S1 File), and indicated by the following significance codes: *** = *p*-value < .0001; ** = *p*-value ≤ .001; * = *p*-value ≤ .01; ■ = *p*-value ≤ .05.

Statistical testing revealed significant differences in peptide hydropathy between protocols (Figs 7 and 8). GRAVY scores were calculated for the entire brain proteome (*n* = 15,049; mean [SD]: –0.382 [0.392]) and for all peptides retrieved across the experiment. While only 13.0% of the total brain proteome is hydrophobic, an average of 39.6% [0.06] of recovered peptides were hydrophobic, suggesting a preferential preservation of hydrophobic domains. In contrast, peptides corresponding to proteins identified by all protocols exhibited lower-than-expected mean hydropathy values, indicating that more hydrophilic domains were preferentially retrieved (Table S5 in S1 File). However, only the low-performance protocol, SDS with FASP, showed a statistically significant deviation (–0.600 [0.863], *p* = 8.1 × 10 ⁻ ^3^; Table S6 in S1 File), and 90% (9/10) of significant pairwise comparisons involved SDS with FASP, suggesting it was an outlier in this regard. Meanwhile, urea-based in-solution methods exhibited the most positive estimated effects (*E*) on GRAVY scores, indicating that they extracted relatively more hydrophobic peptides than other reagents or clean-up strategies (e.g., urea with SPE + iST *vs*. SL: *E* = 0.196, *p* = 3.4 × 10 ⁻^2^).

**Fig 9:**
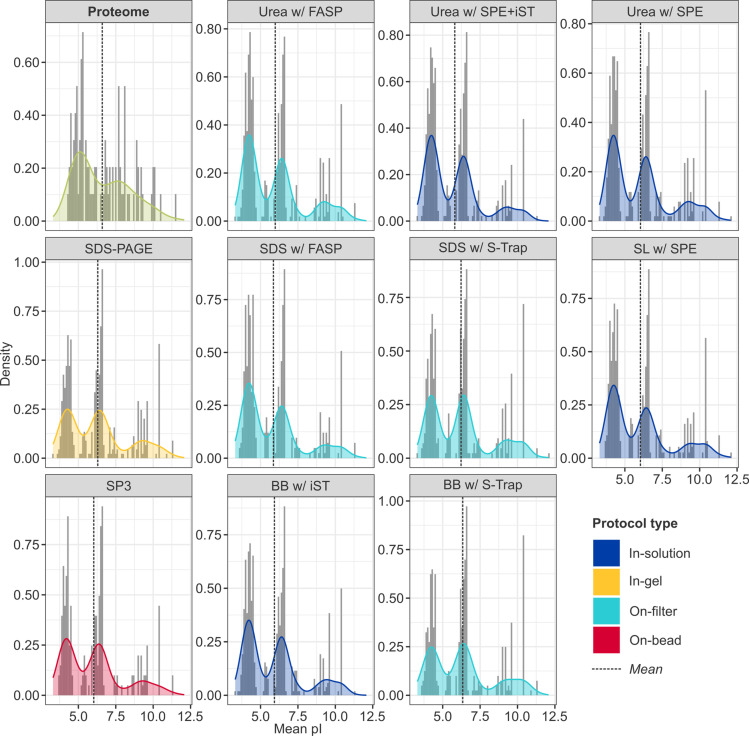
Distribution of isoelectric point by protocol. Distribution of mean pI calculated at the protein-level for proteins retrieved in all protocols (the “common proteome”, *green*), and at the peptide-level for proteins recovered by each protocol, with smoothed density curves. Given the variability in sample sizes, data has been normalised to reflect relative frequency rather than absolute counts (note the differing *y*-axes) and facilitate visual pairwise comparison of extraction buffers (broadly, horizontal rows; e.g., urea, *top*) and clean-up strategies (broadly, vertical columns; e.g., SPE, *right*). Dashed lines reflect the mean pI: a higher value indicates more basic peptides, and a lower value more acidic peptides.

**Fig 10:**
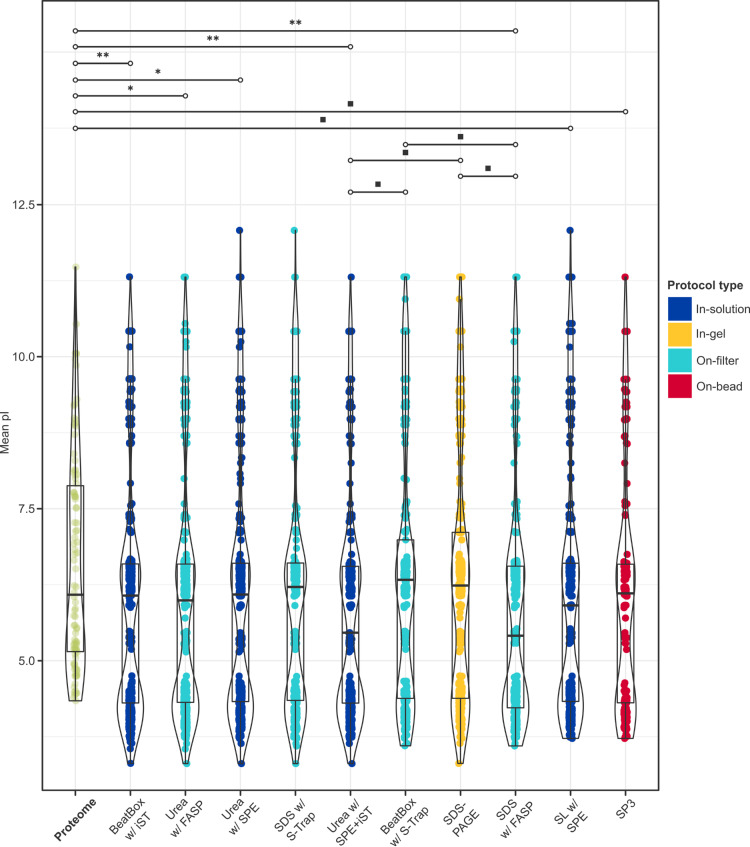
Isoelectric profiles retrieved with each protocol. Results of a linear mixed effects model with Satterthwaite *t*-tests (*n*_obs_ = 4,383) and *post-hoc* pairwise comparisons (*n* = 55) with Bonferroni correction at 95% CI with respect to mean pI, calculated at the protein-level for proteins retrieved in all protocols (the “common proteome”, *green*), and at the peptide-level for proteins recovered by each protocol (from left to right, highest to lowest total proteins). Only statistically significant comparisons are shown (for all other comparisons, see Table S8 in S1 File), and indicated by the following significance codes: *** = *p*-value < .0001; ** = *p*-value ≤ .001; * = *p*-value ≤ .01; ■ = *p*-value ≤ .05.

Analogous statistical testing of the average pI revealed that, like hydropathy, pI of recovered peptides was lower than expected for the common pool (Figs 9 and 10), suggesting that more acidic peptides were preferentially retrieved (Table S7 in S1 File). This relationship was statistically significant for all in-solution protocols (e.g., urea with SPE + iST: *p* = 3.0 x 10^-4^), and for on-filter methods with FASP (e.g., urea with FASP: *p* = 8.1 x 10^-3^; Tables S8 in S1 File). Although the common proteome exhibited the expected bimodal distribution in pI for a eukaryotic proteome [69], a shift toward lower values was apparent: while the major peak associated with cytoplasmic proteins occurred as expected at ~5.0, the major peak corresponding to integral membrane proteins was shifted from ~8.5 to ~7.5 [70]. By contrast, all protocols evidenced a distinct trimodal distribution, with major peaks at ~4.5 and 6.5, and a minor peak ~9.0, in descending order of density (with the exception of BeatBox with S-Trap, whose peak at ~6.5 marginally exceeded that at ~4.5; 0.266 *vs.* 0.248). Corresponding to nuclear proteins [71], this minor peak evidenced the greatest density in urea-based protocols (e.g., urea with SPE: 0.080), corroborating the observation of GO functional enrichment for the mitochondrial compartment with this lysis buffer.

**Fig 11:**
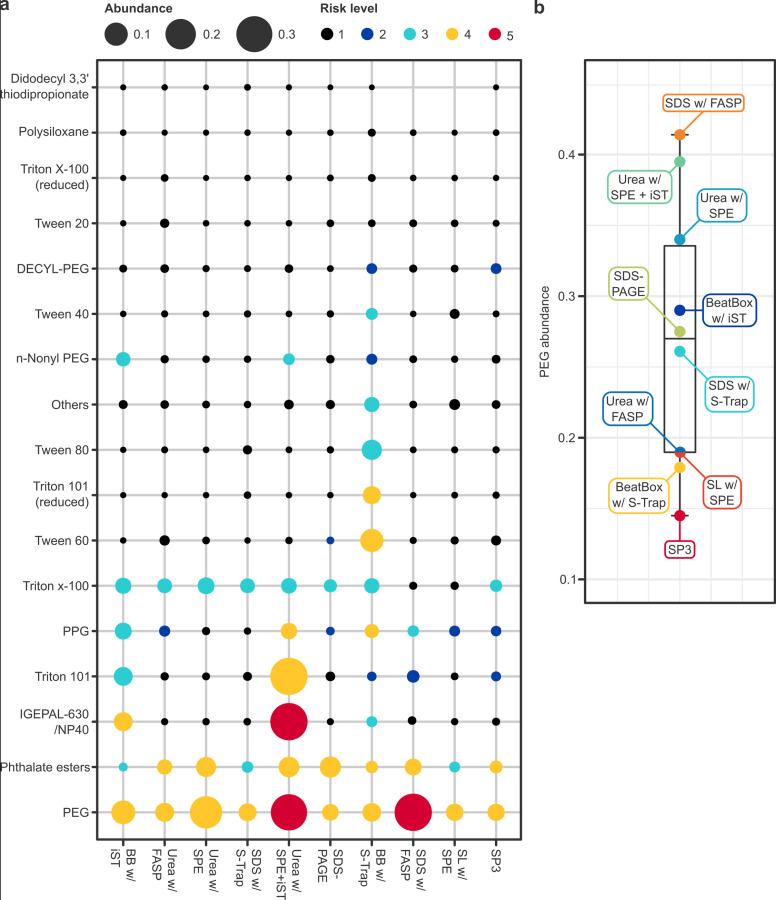
Abundance of common laboratory contaminants. **(a)** Dotplot of contaminants (*y*-axis) identified by protocol (*x*-axis; from left to right, highest to lowest total proteins). Point size indicates total abundance and point colour indicates risk level, which are designated *very high* (5), *high* (4), *medium* (3), *low* (2) and *very low* (1). **(b)** Boxplot of PEG abundance by protocol.

Common laboratory contaminants identified with associated risk levels are illustrated in Fig 11a. Global risk level was very low (1) in nine of ten protocols, and low (2) in BeatBox with S-Trap. Kit-based protocols (i.e., those involving BeatBox and/or iST technology) evidenced a higher abundance of more diverse contaminants, particularly anionic surfactants such as Triton, Tween and IGEPAL-630/NP40. Polyethylene glycol (PEG) was the most abundant contaminant detected at high or very high risk levels (≥ 4) in all samples, posing the greatest risk in the SDS with FASP and urea with SPE + iST protocols. However, no patterns could be discerned in terms of the abundance of PEG contamination and the use of particular extraction buffers or clean-up methods (Fig 11b); nor with the frequency of heating, vortexing, shaking, centrifugation, sonication, or homogenisation steps.

### Limitations

Our study was conducted on ten equal-sized subsamples from a single brain biopsy. Given the destructive nature of the analysis and irreplaceable nature of the material, we elected not to perform replicate extractions: this decision is in line with ethical considerations regarding the treatment of human remains in archaeology, which stipulate a reduced program of sampling for proof-of-concept studies where the feasibility and/or applicability of a method may be questionable [72,73]. Our approach minimised biological variability and provided the internal consistency necessary to ensure that we could effectively compare the efficacy and efficiency of the tested protocols; however, we were unable to assess technical variability. Building on this proof-of-concept study, further work should incorporate both biological and technical replicates to test the generalisability of our findings to a broader population and context.

## Discussion

Only a fraction of the *in vivo* proteome is expected to persist in the archaeological record, and those proteins that survive diagenesis to be extracted, analysed and identified are generally mineralised, highly abundant, and/or possess particular structural features [74]. In this work, we address the absence of a sensitive and specific protocol in palaeoproteomics for non-mineralised tissues. We assessed the efficacy, efficiency and suitability of extraction buffers and clean-up strategies with varied chemistries using archaeological human brain tissues, and describe an optimised LC-FAIMS-MS/MS workflow yielding the largest, most diverse palaeoproteome recovered from any archaeological or fossil material to-date. Further, although 50 mg starting material was employed for extraction, just 5% was injected into the LC-MS platform; demonstrating that even smaller amounts of tissue could be sufficient for deep palaeoproteomic analysis.

We found that urea-based methods (both in-solution and on-filter) proved the most effective and efficient overall, evidencing low contamination and high numbers of protein identifications with relatively high intensities and sequence coverage. In addition, our results suggested that urea-based methods facilitated the recovery of a broader range of peptides, thereby providing greater access to the full diversity of the palaeoproteome: specifically, a greater proportion of non-tryptic and hydrophobic peptides corresponding to membrane proteins, which are more abundant in soft than hard tissues [20]. A chaotropic agent, urea denatures proteins by disrupting non-covalent interactions (such as hydrogen bonds and van der Waals forces), decreasing the net hydrophobic effect of hydrophobic regions and forcing unfolded proteins into solution. This same action destabilises lipid bilayers (compromising membrane integrity and liberating intracellular components) and protein aggregates, which accumulate in aged organs like the brain [75], heart [76], and kidneys [77], and which have previously been identified in preserved soft tissues [21,78]. Indeed, with the exception of SDS-PAGE, urea-based methods were the only to enrich primarily for recovery of the intracellular mitochondrial ATP synthase complex, a critical component of energy production in soft tissues [79], which possess metabolic rates many times that of mineralised tissues [80]. While further work will be required to confirm its suitability for a wider range of samples (see Limitations), urea lysis is likely to be more effective for archaeological soft tissues than those used conventionally in palaeoproteomics for mineralised tissues, such as weak acids or chelating agents [13].

Across the experiment, we identified preferential preservation of hydrophobic proteins, but retrieval of relatively acidic (low-pI) peptides. Protein extraction is strongly influenced by hydropathic and isoelectric behaviour, both of which govern solubility [81]; it is likely that molecular decomposition follows similar principles. During soft tissue decay, the breakdown of proteins and other biomolecules generates acidic byproducts – such as lactic acid, fatty acids, and negatively charged amino acids like aspartate and glutamate – which lower the pH of the surrounding tissue [82]. Under these conditions, hydrophobic proteins, being largely insoluble, are more likely to persist than their hydrophilic counterparts, as their tendency to precipitate and aggregate dominates in all but the most alkaline environments [83]. Consequently, hydrophobic components are notoriously challenging to solubilise and extract [84,85]. While this alone may explain the observed bias toward more soluble, low-pI peptides in our dataset, our pI analyses suggest an additional influence. Proteome-wide distribution studies of pI [69,70,86] reveal an “acidic shoulder” (~ pI 4.75) adjacent to the major acidic peak, which is attributed to an over-representation of low-pI cytoplasmic and extracellular proteins [69]. Cytoskeletal proteins, in particular, are extremely acid-biased, with a clear low-pI shift and trimodal distribution, in contrast to the bimodal distribution observed in cytoplasmic proteins from the same subcellular compartment [69]. This difference is thought to arise from the polymeric nature of cytoskeletal proteins, which precludes true solubility [69] (although progressive changes in solubility have been noted *postmortem* [87,88]). In our dataset, which features an overrepresentation of cytoskeletal proteins, the major peak aligns with this acidic shoulder, suggesting that these recalcitrant fibres contribute to both the preservation and retrieval biases observed.

In step with current best practices in palaeoproteomics [4,6], our study employed stringent search parameters – such as requiring > 2 unique peptides for an identification – to ensure the authenticity of ancient proteins. This stipulation works well for archaeological tissues like bone, skin and hair, where contamination with modern collagens and keratins (which it is essential to minimise but difficult to eliminate [6]) risks misclassifying exogenous peptides as ancient [89]. However, it is likely that for archaeological internal organs, where the risk of a false-positive of this nature is much lower, this stringency leads to more false negatives: that is, discarding genuine identifications that may be better authenticated by other means (e.g., use of post-processing classifiers).

Beyond the risk of false negatives, a greater concern is that genuine identifications may be missed altogether if (semi-)tryptic peptides from digested proteins compete with highly degraded, non-tryptic peptides or contaminants for fragmentation and ionisation. In this respect, the sensitivity of the MS system employed is crucial: the Orbitrap^TM^ Ascend features a number of improvements to the Tribrid^TM^ architecture (including a second ion-routing multipole, which permits parallel ion injection) that together increase the scan speed and sensitivity of the instrument, producing a greater number of higher-quality tandem mass spectra [90]. However, this enhanced sensitivity could also elevate chemical noise, particularly if smaller and/or degraded molecules – such as lipids, metabolites, or other non-peptide contaminants – are transmitted more efficiently into the mass spectrometer.

To address these challenges, we found that LC-FAIMS-MS/MS improves unique protein identification by up to 40%, and represents an effective means of “cleaning” dirty archaeological samples, regardless of the extraction buffer or clean-up strategy used upstream. By filtering out singly charged ions associated with chemical noise, FAIMS both improved the signal-to-noise ratio in complex samples and enabled the detection of low-abundant analytes that would otherwise have been masked, overlapping in mass-to-charge with interfering ions. Moreover, we demonstrate that FAIMS is a highly tuneable technique: given compensation voltages can be optimised to select for ions with specific mobility characteristics, it is likely to be broadly useful for improving protein recovery not only from a range of ancient tissues, but even from a range of depositional environments. Given > 90% of fragment ion spectra from ancient samples fail to be assigned [91], further work in optimising collision energies – or indeed, precursory separation by LC – is expected to help tackle this problem for palaeoproteomics, particularly if coupled with FAIMS.

In sum, this study aimed to identify a suitable method for extracting ancient proteins from soft tissues, maximising the amount of biological information recoverable, and laying the groundwork for future research in non-mineralised bioarchaeological remains. As this method generates increasingly large datasets, optimising their management and analysis presents a new and exciting challenge for palaeoproteomics.

## Supporting information

S1 File Supporting information, including methodological detail, and additional figures and tables. (DOCX)

S1 Dataset Supporting data and analyses. (XLSX)
