# Supplementary material for: Deep palaeoproteomic profiling of archaeological human brains
Source: PLoS One. 2025 May 28;20(5):e0324246. doi: 10.1371/journal.pone.0324246 (PMC12118856; doi:10.1371/journal.pone.0324246)
Supplement: S1 File — (DOCX) [file pone.0324246.s001.docx]

Deep palaeoproteomic profiling of archaeological human brains

Alexandra L. Morton-Hayward^1,2*^, Sarah Flannery^2^, Iolanda Vendrell^2^, & Roman Fischer^2^

*^1^ Department of Earth Sciences, University of Oxford, Oxford, UK*

*^2^ Target Discovery Institute, Nuffield Department of Medicine, University of Oxford, Oxford, UK*

** Corresponding author. Email: alexandra.morton-hayward@earth.ox.ac.uk*

# Methods

## In-solution

### PreOmics® BeatBox iST

| **Component** | **Description** |
| --- | --- |
| DIGEST | Trypsin/LysC mix to digest proteins. |
| RESUSPEND | Reconstitutes lyophilized proteolytic enzymes. |
| LYSE | Denatures, reduces and alkylates proteins. |
| STOP | Stops enzymatic activity. |
| WASH 1 | Cleans peptides from hydrophobic contaminants. |
| WASH 2 | Cleans peptides from hydrophilic contaminants |
| ELUTE | Elutes peptides from the cartridge. |
| LC-LOAD | Loads peptides on reversed-phase LC-MS column. |

1. Transfer 50 mg of brain to a 2 mL collection tube. Add 50 mg glass beads to the sample.
2. Add 100 µL LYSE. Shear the sample by BeatBox sonication for 10 cycles of 30 sec.
3. Place the sample in a heating block at 95 °C and 1,000 rpm for 10 min.
4. Centrifuge at 20 °C and 300 rcf for 10 sec.
5. Add 210 µL RESUSPEND to DIGEST. Shake at 20 °C and 500 rpm for 10 min; pipette up and down to mix.
6. Add 50 µL DIGEST to the tube and place in a preheated heating block at 37 °C and 500 rpm for 3 hr.
7. Add 100 µL STOP to the tube. Shake at 20 °C and 500 rpm for 1 min; pipette up and down to mix.
8. Centrifuge at 16,000 rcf for 1 min.
9. Use the iST adapter (PreOmics) to place the iST cartridge (PreOmics) in a fresh 2 mL waste tube.
10. Transfer the supernatant to the cartridge.
11. Centrifuge at 20 °C and 4,000 rcf for 1 min, or until all the liquid has passed through.
12. Add 200 µL WASH 1 to the cartridge, and repeat step 11.
13. Add 200 µL WASH 2 to the cartridge, and repeat step 11. (Optional: To assess sample loss, transfer the flow-through to a new tube and store at –20 °C prior to analysis.)
14. Use the adapter to place the cartridge in a fresh 1.5 mL collection tube.
15. Add 100 µL ELUTE to the cartridge, and repeat step 11.
16. Repeat step 15.
17. Discard the cartridge and dehydrate the sample in a vacuum concentrator at 45 °C. Store eluted peptides at –20 °C prior to analysis.
18. For LC-MS analysis, add 50 µL LC-LOAD to the collection tube.
19. Shake the collection tube at 20 °C and 500 rpm for 5 min.

### Urea with SPE

| **Reagent** | **Preparation** |
| --- | --- |
| Urea lysis buffer | For 10 mL total volume, combine 4.8 g 8 M urea (NH_2_CONH_2_; Sigma-Aldrich) and 1 mL 1 M TEAB (triethylammonium bicarbonate [C_7_H_17_NO_3_]; Sigma-Aldrich) with 9 mL high performance LC (HPLC)-grade H_2_O. Vortex to mix. |
| 0.5 M TCEP | For 300 μL total volume, dissolve 43.0 mg TCEP (tris[2-carboxyethyl]phosphine [C_9_H_15_O_6_P]; Sigma-Aldrich) in 300 μL HPLC-grade H_2_O. Vortex to mix. |
| 0.5 M IAA | For 300 μL total volume, dissolve 27.8 mg IAA (iodoacetamide [ICH_2_CONH_2_]; Sigma-Aldrich) in 30 μL 100 mM TEAB with 270 μL HPLC-grade H_2_O. Vortex to mix and store at 4 °C until use. |
| 50 mM TEAB | For 10 mL total volume, combine 500 μL 1 M TEAB and 9.5 mL HPLC-grade H_2_O. Vortex to mix. |
| Buffer A (0.1%TFA, 2% ACN) | For 10 mL total volume, combine 10 μL TFA (trifluoroacetic acid [C_2_HF_3_O_2_]; Sigma-Aldrich) and 200 μL ACN (acetonitrile [C_2_H_3_N]; Sigma-Aldrich) with 9.8 mL HPLC-grade H_2_O. Vortex to mix. |
| Buffer B (0.1% TFA, 65% ACN) | For 10 mL total volume, combine 10 μL TFA and 6.5 mL ACN with 3.5 mL HPLC-grade H_2_O. Vortex to mix. |

1. Transfer 50 mg of brain to a 2 mL hard tissue grinding Precellys® lysing tube (Bertin Technologies).
2. Add 200 µL urea lysis buffer.
3. Use the Precellys® 24 Touch homogeniser to lyse at 5,500 rpm for 30 sec. Repeat twice for a total of 3 cycles, cooling on ice between cycles.
4. Incubate at 20 °C and 1,000 rpm for 30 min.
5. Add 4 µL 0.5 M TCEP for a final concentration of 10 mM TCEP.
6. Add 20.4 µL 0.5 M IAA for a final concentration of 50 mM IAA. Incubate at 20 °C and 1,000 rpm for 30 min.
7. Add 1042 µL 50 mM TEAB for a final concentration of 1.5 M urea.
8. Add 1 µg MS-grade trypsin (Promega). Incubate at 37 °C and 1,250 rpm overnight.
9. Add 10.4 µL TFA for a final concentration of 1%. Vortex briefly to mix.
10. Pipette the supernatant into a new 2 mL Eppendorf Protein LoBind® tube (Thermo Fisher Scientific).
11. Centrifuge at 20 °C and 22,000 rcf for 10 min.
12. Pipette the supernatant into a 10 mL Corning® centrifuge tube (Sigma-Aldrich) of buffer A. Vortex briefly to mix.
13. Insert a SOLA^TM^ SPE C_18_ cartridge (Thermo Fisher Scientific) into a new 2 mL waste tube.
14. Add 500 µL buffer B to activate the cartridge, plunging the syringe to filter liquid through the column. Ensure that the C_18_ membrane does not dry out.
15. Add 1 mL buffer A to equilibrate the cartridge, plunging the syringe to filter liquid through the column. Ensure that the C_18_ membrane does not dry out. Dispose of the flow-through.
16. Slowly load the sample onto the cartridge, 1 mL at a time, plunging the syringe to filter liquid through the column. Ensure that the C_18_ membrane does not dry out. Dispose of the flow-through.
17. Wash with 1 mL buffer A, plunging the syringe to filter liquid through the column. Ensure that the C_18_ membrane does not dry out. Dispose of the flow-through and tube. (Optional: To assess sample loss, transfer the flow-through to a new tube and store at –20 °C prior to analysis.)
18. Transfer the cartridge to a new 1.5 mL Eppendorf Protein LoBind® tube (Thermo Fisher Scientific).
19. Add 100 µL buffer B to the cartridge to elute peptides, plunging the syringe to filter all the liquid through the column. Discard the cartridge.
20. Dehydrate in a vacuum concentrator at 30 °C. Store eluted peptides at –20 °C prior to analysis.

### Urea with SPE + iST

| **Reagent** | **Preparation** |
| --- | --- |
| Urea lysis buffer | For 10 mL total volume, combine 4.8 g 8 M urea and 1 mL 1 M TEAB with 9 mL HPLC-grade H_2_O. Vortex to mix. |
| 0.5 M IAA | For 300 μl total volume, combine 27.8 mg IAA and 30 μL 100 mM TEAB with 270 μL HPLC-grade H_2_O. Vortex to mix and store at 4 °C until use. |
| 50 mM TEAB | For 10 mL total volume, combine 500 μL 1 M TEAB and 9.5 mL HPLC-grade H_2_O. Vortex to mix. |
| Buffer A (0.1% TFA, 2% ACN) | For 10 mL total volume, combine 10 μL TFA and 200 μL ACN with 9.8 mL HPLC-grade H_2_O. Vortex to mix. |
| Buffer B (0.1% TFA, 65% ACN) | For 10 mL total volume, combine 10 μL TFA and 6.5 mL ACN with 3.5 mL HPLC-grade H_2_O. Vortex to mix. |
| Buffer C (0.1% TFA, 50% ACN) | For 1 mL total volume, combine 1 μL TFA and 500 μL ACN with 499 μL HPLC-grade H_2_O. Vortex to mix. |
| WASH 1 | Cleans peptides from hydrophobic contaminants. |
| WASH 2 | Cleans peptides from hydrophilic contaminants |
| ELUTE | Elutes peptides from the cartridge. |
| LC-LOAD | Loads peptides on reversed-phase LC-MS column. |

Complete steps 1-18 as for the protocol *Urea with SPE*, and proceed as follows:

1. Reconstitute the sample by adding 100 µL buffer A. Sonicate at 4 °C for 5 min.
2. Incubate at 20 °C and 1,250 rpm for 5 min.
3. Insert the iST cartridge into a new 2 mL waste tube. Add 200 µL buffer C to activate the cartridge. Centrifuge at 20 °C and 4,000 rcf for 1 min, or until all the liquid has passed through.
4. Add 200 μL buffer A to equilibrate the cartridge. Centrifuge at 20 °C and 4,000 rcf for 1 min, or until all the liquid has passed through. Dispose of the flow-through.
5. Add 50 μL of sample and 150 μL buffer A to the cartridge.
6. Centrifuge at 20 °C and 4,000 rcf for 1 min, or until all the liquid has passed through.
7. Add 200 µL WASH 1 to the cartridge, and repeat step 6.
8. Add 200 µL WASH 2 to the cartridge, and repeat step 6. Dispose of the flow-through. (Optional: To assess sample loss, transfer the flow-through to a new tube and store at –20 °C prior to analysis.)
9. Transfer the iST cartridge to a new 1.5 mL collection tube.
10. Add 100 µL ELUTE to the cartridge, and repeat step 6.
11. Repeat step 10.
12. Discard the cartridge and dehydrate the sample in a vacuum concentrator at 45 °C. Store eluted peptides at –20 °C prior to analysis.
13. For LC-MS analysis, add 50 µL LC-LOAD to the collection tube.
14. Shake the collection tube at 20 °C and 500 rpm for 5 min.

### Sodium laurate with SPE

| **Reagent** | **Preparation** |
| --- | --- |
| 0.5 M TCEP | For 300 μL total volume, dissolve 43.0 mg TCEP in 300 μL HPLC-grade H_2_O. Vortex to mix. |
| 0.5 M IAA | For 300 μL total volume, combine 27.8 mg IAA and 30 μL 100 mM TEAB with 270 μL HPLC-grade H_2_O. Vortex to mix and store at 4 °C until use.. |
| 50 mM TEAB | For 10 mL total volume, combine 500 μL 1 M TEAB and 9.5 mL HPLC-grade H_2_O. Vortex to mix. |
| 100 mM TEAB | For 10 mL total volume, combine 1 mL 1 M TEAB and 9 mL HPLC-grade H_2_O. Vortex to mix. |
| 1% sodium laureate | For 1 mL total volume, combine 10 μg lauric acid (C_12_H_24_O_2_; Sigma-Aldrich) and 1 mL of HPLC-grade H_2_O. Vortex to mix. |
| Buffer A (0.1% TFA, 2% ACN) | For 10 mL total volume, combine 10 μL TFA and 200 μL ACN with 9.8 mL HPLC-grade H_2_O. Vortex to mix. |
| Buffer B (0.1% TFA, 65% ACN) | For 10 mL total volume, combine 10 μL TFA and 6.5 mL ACN with 3.5 mL HPLC-grade H_2_O. Vortex to mix. |

1. Transfer 50 mg of brain to a 2 mL hard tissue grinding Precellys® lysing tube (Bertin Technologies).
2. Add 50 µL 1% sodium laurate.
3. Use the Precellys® 24 Touch homogeniser (Bertin Technologies) to lyse at 5,500 rpm for 30 sec. Repeat twice for a total of 3 cycles, cooling on ice between cycles.
4. Sonicate at 4 °C for 10 min.
5. Vortex for 1 min.
6. Add 1 µL 0.5 M TCEP for a final concentration of 10 mM.
7. Add 2 µL 0.5 M IAA for a final concentration of 20 mM. Incubate at 20 °C for 30 min.
8. Add 97 µL 1% sodium laurate for 150 µL total volume.
9. Add 1 µg MS-grade trypsin (Promega). Incubate at 37 °C and 1,000 rpm for 3 hr.
10. Add 1.5 µL TFA to a final concentration of 1%.
11. Centrifuge at 20 °C and 22,000 rcf for 10 min. Transfer the supernatant to a new 2 mL Eppendorf Protein LoBind® tube (Thermo Fisher Scientific). Note the volume recovered.
12. Add a volume of ethyl acetate equal to the volume of supernatant recovered, and vortex for 1 min.
13. Centrifuge at 15,700 rcf for 10 min.
14. Transfer lower phase to new Eppendorf Protein LoBind® tube (Thermo Fisher Scientific).
15. Repeat steps 12-14.
16. Insert a SPE C_18_ cartridge into a new 2 mL waste tube.
17. Add 500 µL buffer B to activate the cartridge, plunging the syringe to filter liquid through the column. Ensure that the C_18_ membrane does not dry out.
18. Add 1 mL buffer A to equilibrate the cartridge, plunging the syringe to filter liquid through the column. Ensure that the C_18_ membrane does not dry out. Dispose of the flow-through.
19. Load the sample onto the cartridge.
20. Wash with 1 mL buffer A, plunging the syringe to filter liquid through the column. Ensure that the C_18_ membrane does not dry out. Dispose of the flow-through and tube. (Optional: To assess sample loss, transfer the flow-through to a new tube and store at –20 °C prior to analysis.)
21. Transfer the cartridge to a new 1.5 mL Eppendorf Protein LoBind® tube (Thermo Fisher Scientific).
22. Add 100 µL buffer B to the cartridge to elute peptides, plunging the syringe to filter all the liquid through the column. Repeat and discard the cartridge.
23. Dehydrate in a vacuum concentrator at 30 °C. Store eluted peptides at –20 °C prior to analysis.

## In-gel

### SDS-PAGE

| **Reagent** | **Preparation** |
| --- | --- |
| 5% SDS lysis buffer | For 1 mL total volume, combine 250 µL 20% SDS (sodium dodecyl sulphate [C_12_H_25_NaO_4_S]; Sigma-Aldrich) and 100 µL 100 mM TEAB with 650 µL HPLC-grade H_2_O. Vortex to mix. |
| NuPAGE^TM^ LDS (4x) sample buffer (Invitrogen^TM^, Thermo Fisher Scientific) | A basic solution (pH 8.5) mixed in a 1:3 ratio with the sample that stains the moving ion front for PAGE. Contains 141 mM Tris (Tris[hydroxymethyl]aminomethane [NH_2_C(CH_2_OH)_3_]), 106 mM Tris-HCl (Tris[hydroxymethyl]aminomethane hydrochloride [NH_2_C(CH_2_OH)_3_•HCl]), 2% LDS (lithium dodecyl sulphate [CH_3_(CH_2_)_11_OSO_3_Li]), 0.51mM EDTA (ethylenediaminetetraacetic acid [(HO_2_CCH_2_)_2_NCH_2_CH_2_N(CH_2_CO_2_H)_2_]), 0.22 mM SERVA^TM^ Blue G-250, and 0.175 mM Phenol Red. |
| NuPAGE^TM^ MES SDS (20x) running buffer (Invitrogen^TM^, Thermo Fisher Scientific) | A neutral solution (pH 7.3) that facilitates ion migration during PAGE. Contains 50 mM MES (2-[n-morpholino]ethanesulfonic acid [C_6_H_13_NO_4_S]), 50 mM Tris, 0.1% SDS, and 1 mM EDTA. |
| 5% acetic acid | For 20 mL total volume, combine 10 mL methanol, 1 mL glacial acetic acid (CH_3_CO_2_H; Sigma-Aldrich) and 9 mL HPLC-grade H_2_O. Vortex to mix and store at 4 °C until use. |
| 100 mM ambic | For 1 mL total volume, dissolve 7.9 mg ambic (ammonium bicarbonate [NH_4_HCO_3_]; Sigma-Aldrich) in 1 mL HPLC-grade H_2_O. Vortex to mix and vacuum-filter with a 0.22 µm filter. |
| 200 mM DTT | For 1 mL total volume, dissolve 30.9 mg DTT (dithiothreitol [C_4_H_10_O_2_S_2_]; Sigma-Aldrich) in 1 mL HPLC-grade H_2_O. Vortex to mix and store at 4 °C until use. |
| 10 mM DTT | For 1 mL total volume, combine 50 µL 200 mM DTT with 950 µL 100 mM ambic. Vortex to mix and store at 4 °C until use. |
| 50 mM IAA | For 1 mL total volume, dissolve 9.3 mg IAA in 1 mL HPLC-grade H_2_O. Vortex to mix and store at 4 °C until use. |
| 50 mM ambic | For 1 mL total volume, combine 500 µL 100 mM ambic and 500 µL HPLC-grade H_2_O. Vortex to mix and vacuum-filter with a 0.22 µm filter. |
| Digestion buffer | For 200 µL total volume, combine 200 µL 50 mM ambic with 1 µg trypsin. Pipette gently to mix. |
| Buffer A (5% FA, 50% ACN) | For 10 mL total volume, combine 500 µL FA (formic acid [CH_2_O_2_]; Sigma-Aldrich) and 5 mL ACN with 4.5 mL HPLC-grade H_2_O. Vortex to mix. |
| Buffer B (5% FA, 85% ACN) | For 10 mL total volume, combine 500 µL FA and 8.5 mL ACN with 1 mL HPLC-grade H_2_O. Vortex to mix. |

1. Transfer 50 mg of brain to a 2 mL hard tissue grinding Precellys® lysing tube.
2. Add 100 µL 5% SDS lysis buffer.
3. Use the Precellys® 24 Touch homogeniser to lyse at 5,500 rpm for 30 sec. Repeat twice for a total of 3 cycles, cooling on ice between cycles.
4. Centrifuge at 20 °C and 4,000 rcf for 5 min.
5. Pipette the supernatant into a 2 mL LoBind® tube. Centrifuge at 20 °C and 22,000 rcf for 5 min.
6. Pipette the supernatant into a new 2 mL collection tube. Add 33.3 µL NuPAGE^TM^ LDS (4x) sample buffer (Invitrogen^TM^, Thermo Fisher Scientific).
7. Add 3.3 µL 200 mM DTT to a final concentration of 1%.
8. Incubate at 20 °C and 2,000 rpm for 1 hr.
9. Centrifuge at 20 °C and 22,000 rcf for 2 min.
10. Insert a 1 mm NuPAGE^TM^ Bis-Tris gel (Invitrogen^TM^, Thermo Fisher Scientific) into a Mini Gel Tank (Invitrogen^TM^, Thermo Fisher Scientific) and load with NuPAGE^TM^ MES SDS (20x) running buffer (Invitrogen^TM^, Thermo Fisher Scientific). Remove and discard the comb.
11. Load 20 µL of sample into each of six gel lanes.
12. Cover the chamber and connect the electrodes. Run the gel at a constant voltage of 120 V for 10 min.
13. Disconnect the electrodes and remove the cover. Remove the gel and place in a sterile petri dish. Cover the gel with InstantBlue^TM^ staining dye (Sigma-Aldrich), and place in a rocker for 10 min.
14. Wash the gel of the dye with HPLC-grade H_2_O.
15. Using a sterile scalpel, excise each gel lane and cut into approximately 1 mm^2^ cubes. Transfer each lane to a new 1.5 mL LoBind® tube and add 1 µL 5% acetic acid.
16. Incubate at 20 °C and 1,000 rpm overnight.
17. Remove the supernatant from the gel pieces.
18. Add 200 µL ACN and vortex. Allow to dehydrate at 20 °C for 5 min.
19. Remove the supernatant and repeat step 18.
20. Add 30 µL 10 mM DTT and incubate at 20 °C for 30 min.
21. Remove the supernatant and add 30 µL 50 mM IAA. Incubate at 20 °C for 30 min.
22. Remove the supernatant and add 200 µL ACN. Allow to dehydrate at 20 °C for 5 min.
23. Remove the supernatant and add 200 µL 100 mM ambic. Allow to rehydrate at 20 °C for 10 min.
24. Remove the supernatant and add 200 µL ACN. Allow to dehydrate at 20 °C for 5 min.
25. Add 100 µL digestion buffer. Place the tubes on ice with shaking at 500 rpm for 10 min.
26. Centrifuge at 20 °C and 4,000 rcf for 5 sec.
27. Remove the supernatant and add 15 µL 50 mM ambic.
28. Incubate at 37 °C overnight.
29. Add 50 µL of 50 mM ambic and incubate at 20 °C for 10 min, gently vortexing every 2-3 min.
30. Transfer the supernatant to a new 1.5 mL LoBind® tube.
31. Add 50 µL of buffer A to the gel pieces and incubate at 20 °C for 10 min, gently vortexing every 2-3 min.
32. Transfer the supernatant to the collection tube.
33. Add 50 µL of buffer B to the gel pieces and incubate at 20 °C for 10 min, gently vortexing every 2-3 min.
34. Repeat step 32.
35. Combine the gel pieces in a new 1.5 mL LoBind® tube. Dehydrate in a vacuum concentrator at 30 °C. Store eluted peptides at –20 °C prior to analysis.

## On-filter

### PreOmics® BeatBox with S-Trap^TM^ micro

| **Reagent** | **Preparation** |
| --- | --- |
| 0.5 M TCEP | For 300 μL total volume, dissolve 43.0 mg TCEP in 300 μL HPLC-grade H_2_O. Vortex to mix. |
| 5% SDS lysis buffer | For 5 mL total volume, combine 1.25 mL 20% SDS, 100 μL 0.5 M TCEP and 500 μL 100 mM TEAB in 3.15 mL HPLC-grade H_2_O. Vortex to mix. |
| S-Trap^TM^ buffer | For 5 mL total volume, combine 4.5 mL methanol and 500 μL 100 mM TEAB. Vortex to mix. |
| 0.5 M IAA | For a total volume of 300 μL, combine 27.8 mg IAA and 30 μL 100 mM TEAB with 270 μL HPLC-grade H_2_O. Vortex to mix and store at 4 °C until use. |
| 50 mM TEAB | For 10 mL total volume, combine 500 µL 1 M TEAB and 9.5 mL HPLC-grade H_2_O. Vortex to mix. |
| Buffer A (0.2% FA) | For 1 mL total volume, combine 2 μL TFA and 1 mL HPLC-grade H_2_O. Vortex to mix. |
| Buffer B (0.2% FA, 50% ACN) | For 1 mL total volume, combine 2 μL TFA and 500 μL ACN with 498 μL HPLC-grade H_2_O. Vortex to mix. |

1. Transfer 50 mg of brain to a 2 mL collection tube. Add 50 mg glass beads to the sample.
2. Add 1 mL of 5% SDS lysis buffer.
3. Shear the sample by BeatBox sonication for 10 cycles of 30 sec.
4. Centrifuge at 20 °C and 4,000 rcf for 1 min.
5. Transfer the supernatant to a 2 mL LoBind® tube and centrifuge at 20 °C and 22,000 rcf for 10 min.
6. Add 40 μL 0.5 M IAA for a final concentration of 20 mM. Incubate at 20 °C for 30 min.
7. Add 430 μL 12% phosphoric acid for a final concentration of 1.2%.
8. Add 3 mL of S-Trap^TM^ buffer.
9. Slowly load the sample onto the S-Trap^TM^ micro (Protifi, Biosys Technologies, Inc.), 500 μL at a time. After each load, centrifuge at 20 °C and 4,000 rcf for 30 sec, and discard both the filter and flow-through. Insert a new filter and repeat the process until all the sample has been loaded.
10. Wash the S-Trap^TM^ with 400 µL S-Trap^TM^ buffer, and centrifuge at 20 °C and 4,000 rcf for 30 sec. Repeat this step.
11. Transfer the filter to a new 2 mL S-Trap^TM^ tube. Wash and centrifuge twice more in the new tube, for a total of four washes.
12. Transfer the S-Trap^TM^ to a new 1.5 mL S-Trap^TM^ tube. Add 125 µL 50 mM TEAB.
13. Add 1 µg MS-grade trypsin (Promega). Incubate at 37 °C overnight.
14. Add 80 µL 50 mM TEAB.
15. Add 80 µL buffer A. Check pH < 2.0.
16. Add 80 µL buffer B. Discard the S-Trap^TM^ micro.
17. Dehydrate in a vacuum concentrator at 30 °C. Store eluted peptides at –20 °C prior to analysis.

### SDS with S-Trap^TM^ micro

| **Reagent** | **Preparation** |
| --- | --- |
| 0.5 M TCEP | For 300 μL total volume, dissolve 43.0 mg TCEP in 300 μL HPLC-grade H_2_O. Vortex to mix. |
| 5% SDS lysis buffer | For 5 mL total volume, combine 1.25 mL 20% SDS, 100 μL 0.5 M TCEP and 500 μL 100 mM TEAB in 3.15 mL HPLC-grade H_2_O. Vortex to mix. |
| S-Trap^TM^ buffer | For 5 mL total volume, combine 4.5 mL methanol and 500 μL 100 mM TEAB. Vortex to mix. |
| 0.5 M IAA | For a total volume of 300 μL, combine 27.8 mg IAA and 30 μL 100 mM TEAB with 270 μL HPLC-grade H_2_O. Vortex to mix and store at 4 °C until use. |
| 50 mM TEAB | For 10 mL total volume, combine 500 µL 1 M TEAB and 9.5 mL HPLC-grade H_2_O. Vortex to mix. |
| Buffer A (0.2% FA) | For 1 mL total volume, combine 2 μL TFA and 1 mL HPLC-grade H_2_O. Vortex to mix. |
| Buffer B (0.2% FA, 50% ACN) | For 1 mL total volume, combine 2 μL TFA and 500 μL ACN with 498 μL HPLC-grade H_2_O. Vortex to mix. |

1. Transfer 50 mg of brain to a 2 mL hard tissue grinding Precellys® lysing tube.
2. Add 1 mL of 5% SDS lysis buffer.
3. Use the Precellys® 24 Touch homogeniser to lyse at 5,500 rpm for 30 sec. Repeat twice for a total of 3 cycles, cooling on ice between cycles.
4. Centrifuge at 20 °C and 4,000 rcf for 1 min.
5. Transfer the supernatant to a 2 mL LoBind® tube and centrifuge at 20 °C and 22,000 rcf for 10 min.
6. Add 40 μL 0.5 M IAA for a final concentration of 20 mM. Incubate at 20 °C for 30 min.
7. Add 430 μL 12% phosphoric acid for a final concentration of 1.2%.
8. Add 3 mL of S-Trap^TM^ buffer.
9. Slowly load the sample onto the S-Trap^TM^ micro (Protifi, Biosys Technologies, Inc.), 500 μL at a time. After each load, centrifuge at 20 °C and 4,000 rcf for 30 sec, and discard both the filter and flow-through. Insert a new filter and repeat the process until all the sample has been loaded.
10. Wash the S-Trap^TM^ with 400 µL S-Trap^TM^ buffer, and centrifuge at 20 °C and 4,000 rcf for 30 sec. Repeat this step.
11. Transfer the filter to a new 2 mL S-Trap^TM^ tube. Wash and centrifuge twice more in the new tube, for a total of four washes.
12. Transfer the S-Trap^TM^ to a new 1.5 mL S-Trap^TM^ tube. Add 125 µL 50 mM TEAB.
13. Add 1 µg MS-grade trypsin (Promega). Incubate at 37 °C overnight.
14. Add 80 µL 50 mM TEAB.
15. Add 80 µL buffer A. Check pH < 2.0.
16. Add 80 µL buffer B. Discard the S-Trap^TM^ micro.
17. Dehydrate in a vacuum concentrator at 30 °C. Store eluted peptides at –20 °C prior to analysis.

### Urea with FASP

| **Reagent** | **Preparation** |
| --- | --- |
| Urea lysis buffer | For 10 mL total volume, combine 4.8 g 8 M urea and 1 mL 1 M TEAB with 9 mL HPLC-grade H_2_O. Vortex to mix. |
| Buffer A (0.1% TFA, 50% ACN) | For 1 mL total volume, combine 1μL TFA and 500 μL ACN with 499 μL HPLC-grade H_2_O. Vortex to mix. |
| 0.5 M TCEP | For 300 μL total volume, dissolve 43.0 mg TCEP in 300 μL HPLC-grade H_2_O. Vortex to mix. |
| 0.5 M IAA | For 300 μL total volume, combine 27.8 mg IAA and 30 μL 100 mM TEAB with 270 μL HPLC-grade H_2_O. Vortex to mix and store at 4 °C until use. |
| 50 mM TEAB | For 10 mL total volume, combine 500 μL 1 M TEAB and 9.5 mL HPLC-grade H_2_O. Vortex to mix. |
| Buffer B (0.1% TFA) | For 1 mL total volume, combine 1 μL TFA and 1 mL HPLC-grade H_2_O. Vortex to mix. |

1. Transfer 50 mg of brain to a 2 mL hard tissue grinding Precellys® lysing tube.
2. Add 200 µL urea lysis buffer.
3. Use the Precellys® 24 Touch homogeniser to lyse at 5,500 rpm for 30 sec. Repeat twice for a total of 3 cycles, cooling on ice between cycles.
4. Centrifuge at 20 °C and 4,000 rcf for 1 min.
5. Insert a Sartorius Vivacon^TM^ 500 30 kDa MWCO filter (Thermo Fisher Scientific) into a 2 mL adapter tube.
6. Add 200 µL buffer A. Centrifuge at 20 °C and 14,300 rcf for 10 min, or until all the liquid has passed through the filter. Discard the flow-through.
7. Load the sample on the filter. Centrifuge at 20 °C and 14,300 rcf for 10 min, or until all the liquid has passed through. Transfer the flow-through to a 2 mL LoBind® tube.
8. Add 200 µL urea lysis buffer to the filter.
9. Add 4 µL 0.5 M TCEP for a final concentration of 10 mM TCEP.
10. Add 20.4 µL 0.5 M IAA for a final concentration of 50 mM IAA. Incubate at 20 °C for 30 min.
11. Centrifuge at 20 °C and 14,300 rcf for 10 min, or until all the liquid has passed through.
12. Add 200 µL 50 mM TEAB. Centrifuge at 20 °C and 14,300 rcf for 10 min, or until all the liquid has passed through.
13. Repeat step 12 twice, for a total of three washes with 50 mM TEAB. Discard the flow-through.
14. Transfer the filter to a new adapter tube.
15. Add 200 µL 50mM TEAB, and 1 µg MS-grade trypsin (Promega). Incubate at 37 °C overnight.
16. Centrifuge at 20 °C and 14,300 rcf for 10 min to elute peptides. Transfer the flow-through to a 2 mL LoBind® tube and keep on ice.
17. Add 200 µL buffer B to the filter. Centrifuge at 20 °C and 14,300 rcf for 10 min, or until all the liquid has passed through.
18. Add 200 µL buffer A. Centrifuge at 20 °C and 14,300 rcf for 10 min, or until all the liquid has passed through.
19. Combine the flow-through from steps 17 and 18 with that placed on ice in step 16. Discard the filter.
20. Dehydrate in a vacuum concentrator at 30 °C. Store eluted peptides at –20 °C prior to analysis.

### SDS with FASP

| **Reagent** | **Preparation** |
| --- | --- |
| 5% SDS lysis buffer | For 1 mL total volume, combine 250 µL 20% SDS and 100 µL TEAB with 650 µL HPLC-grade H_2_O. Vortex to mix. |
| Buffer A (0.1% TFA, 50% ACN) | For 1 mL total volume, combine 1 μL TFA and 500 μL ACN with 499 μL HPLC-grade H_2_O. Vortex to mix. |
| 0.5 M TCEP | For 300 μL total volume, dissolve 43.0 mg TCEP in 300 μL HPLC-grade H_2_O. Vortex to mix. |
| Urea lysis buffer | For 10 mL total volume, combine 4.8 g 8 M urea and 1 mL 1 M TEAB with 9 mL HPLC-grade H_2_O. Vortex to mix. |
| 0.5 M IAA | For 300 μL total volume, combine 27.8 mg IAA and 30 μL 100 mM TEAB with 270 μL HPLC-grade H_2_O. Vortex to mix and store at 4 °C until use. |
| 50 mM TEAB | For 10 mL total volume, combine 500 μL 1 M TEAB and 9.5 mL HPLC-grade H_2_O. Vortex to mix. |
| Buffer B (0.1% TFA) | For 1 mL total volume, combine 1 μL TFA and 1 mL HPLC-grade H_2_O. Vortex to mix. |

1. Transfer 50 mg of brain to a 2mL hard tissue grinding Precellys® lysing tube (Bertin Technologies).
2. Add 200 µL 5% SDS lysis buffer.
3. Use the Precellys® 24 Touch homogeniser to lyse at 5,500 rpm for 30 sec. Repeat twice for a total of 3 cycles, cooling on ice between cycles.
4. Centrifuge at 20 °C and 3,000 rcf for 1 min.
5. Insert a Sartorius Vivacon^TM^ 500 30 kDa MWCO filter (Thermo Fisher Scientific) into a 2 mL adapter tube.
6. Add 200 µL buffer A. Centrifuge at 20 °C and 14,300 rcf for 10 min, or until all the liquid has passed through the filter. Discard the flow-through.
7. Load the sample on the filter. Centrifuge at 20 °C and 14,300 rcf for 10 min, or until all the liquid has passed through. Transfer the flow-through to a 2 mL LoBind® tube.
8. Add 200 µL urea lysis buffer to the filter.
9. Add 4 µL 0.5 M TCEP for a final concentration of 10 mM TCEP.
10. Add 20.4 µL 0.5 M IAA for a final concentration of 50 mM IAA. Incubate at 20 °C for 30 min.
11. Centrifuge at 20 °C and 14,300 rcf for 10 min, or until all the liquid has passed through.
12. Add 200 µL 50 mM TEAB. Centrifuge at 20 °C and 14,300 rcf for 10 min, or until all the liquid has passed through.
13. Repeat step 12 twice, for a total of three washes with 50 mM TEAB. Discard the flow-through.
14. Transfer the filter to a new adapter tube.
15. Add 200 µL 50mM TEAB, and 1 µg MS-grade trypsin (Promega). Incubate at 37 °C overnight.
16. Centrifuge at 20 °C and 14,300 rcf for 10 min to elute peptides. Transfer the flow-through to a 2 mL LoBind® tube and keep on ice.
17. Add 200 µL buffer B to the filter. Centrifuge at 20 °C and 14,300 rcf for 10 min, or until all the liquid has passed through.
18. Add 200 µL buffer A. Centrifuge at 20 °C and 14,300 rcf for 10 min, or until all the liquid has passed through.
19. Combine the flow-through from steps 17 and 18 with that placed on ice in step 16. Discard the filter.
20. Dehydrate in a vacuum concentrator at 30 °C. Store eluted peptides at –20 °C prior to analysis.

## On-bead

### SP3

| **Reagent** | **Preparation** |
| --- | --- |
| 0.5% SDS | For 1 mL total volume, combine 25 μL 20% SDS and 975 μL HPLC-grade H_2_O. Vortex to mix. |
| 200 mM DTT | For 1 mL total volume, dissolve 30.9 mg DTT in 1 mL HPLC-grade H_2_O. Vortex to mix and store at 4 °C until use. |
| 0.5 M IAA | For 300 μL total volume, combine 27.8 mg IAA and 30 μL 100 mM TEAB with 270 μL HPLC-grade H_2_O. Vortex to mix and store at 4 °C until use. |
| 50 mM TEAB | For 10 mL total volume, combine 500 μL 1 M TEAB and 9.5 mL HPLC-grade H_2_O. Vortex to mix. |
| SP3 beads | For 10 µL total volume, combine 5 µL hydrophobic and 5 µL hydrophilic beads. |

1. Transfer 50 mg of brain to a 2 mL hard tissue grinding Precellys® lysing tube.
2. Add 50 µL 0.5% SDS.
3. Use the Precellys® 24 Touch homogeniser to lyse at 5,500 rpm for 30 sec. Repeat twice for a total of 3 cycles, cooling on ice between cycles.
4. Centrifuge at 20 °C and 22,000 rcf for 5 min. Transfer the supernatant to a new 1.5 mL LoBind® tube.
5. Add 1.25 µL 200 mM DTT to a final concentration of 5 mM. Incubate at 20 °C for 30 min.
6. Add 2.26 µL 0.5 M IAA to a final concentration of 20 mM. Incubate at 20 °C for 30 min.
7. Centrifuge at 20 °C and 22,000 rcf for 5 min.
8. Transfer the supernatant to a new 1 µL LoBind® tube containing 3 µL of SP3 beads in a 1:1 hydrophobic:hydrophilic ratio.
9. Add 125 µL ACN to a final concentration of 70%. Mix at 20 °C and 1,000 rpm for 18 min.
10. Place the sample on the magnetic rack and leave for 2 min, to allow all beads to migrate to the plate.
11. Pipette off and discard the supernatant. Keep the sample on the magnetic rack until steps 12-15 are complete.
12. Rinse with 200 µL 70% ethanol. Leave for 15 sec, then pipette off and discard the supernatant.
13. Rinse with 200 µL 100% ACN. Leave for 15 sec, then pipette off and discard the supernatant.
14. Repeat step 12.
15. Repeat step 13.
16. Remove the tube from the magnetic rack and allow the beads to settle to the bottom.
17. Add 150 µL 50 mM TEAB, or enough of this digestion buffer to ensure all beads are covered.
18. Add 1 µg MS-grade trypsin (Promega). Incubate at 37 °C and 1,000 rpm overnight.
19. Transfer the supernatant to a new 1.5 mL LoBind® tube.
20. Add 1.5 µL TFA to a final concentration of 1%.
21. Dehydrate in a vacuum concentrator at 30 °C. Store eluted peptides at –20 °C prior to analysis.

#

# Figures & Tables

**Table S1.** **DDA and DIA search strategies |** Protein identification and quantification analyses conducted with associated software packages.

| **Data type** | **Search strategy** |
| --- | --- |
| **DIA** | DIA-NN [[v.1.9; 1]](https://paperpile.com/c/6UBX72/ASRA/?prefix=v.1.9%3B) with library-free search. |
|  | DIA-NN [[v.1.9; 1]](https://paperpile.com/c/6UBX72/ASRA/?prefix=v.1.9%3B) with spectral library search, based on library-free generation |
|  | DIA-NN [[v.1.9; 1]](https://paperpile.com/c/6UBX72/ASRA/?prefix=v.1.9%3B) with spectral library search, based on *in silico* generation. |
|  | FragPipe [[v.22.0; 2]](https://paperpile.com/c/6UBX72/Aoxl/?prefix=v.22.0%3B) with spectral deconvolution with DIA-Umpire Signal Extraction [[v.2.0; 3]](https://paperpile.com/c/6UBX72/dH3I/?prefix=v.2.0%3B), and quantification with DIA-NN [[v.1.8.2; 1]](https://paperpile.com/c/6UBX72/ASRA/?prefix=v.1.8.2%3B). |
|  | MaxQuant [[v.2.6.1.0; 4]](https://paperpile.com/c/6UBX72/Os8R/?prefix=v.2.6.1.0%3B) with MaxDIA [[5]](https://paperpile.com/c/6UBX72/KL33) for spectral library search, based on DDA library generation. |
| **DDA** | FragPipe [[v.22.0; 6]](https://paperpile.com/c/6UBX72/bSzI/?prefix=v.22.0%3B) with LFQ-MBR. |
|  | MaxQuant [[v.2.4.3.0; 4]](https://paperpile.com/c/6UBX72/Os8R/?prefix=v.2.4.3.0%3B) with LFQ-MBR and dependent peptides search. |
|  | PEAKS Studio 13 (Bioinformatics Solutions, Inc.). |

**
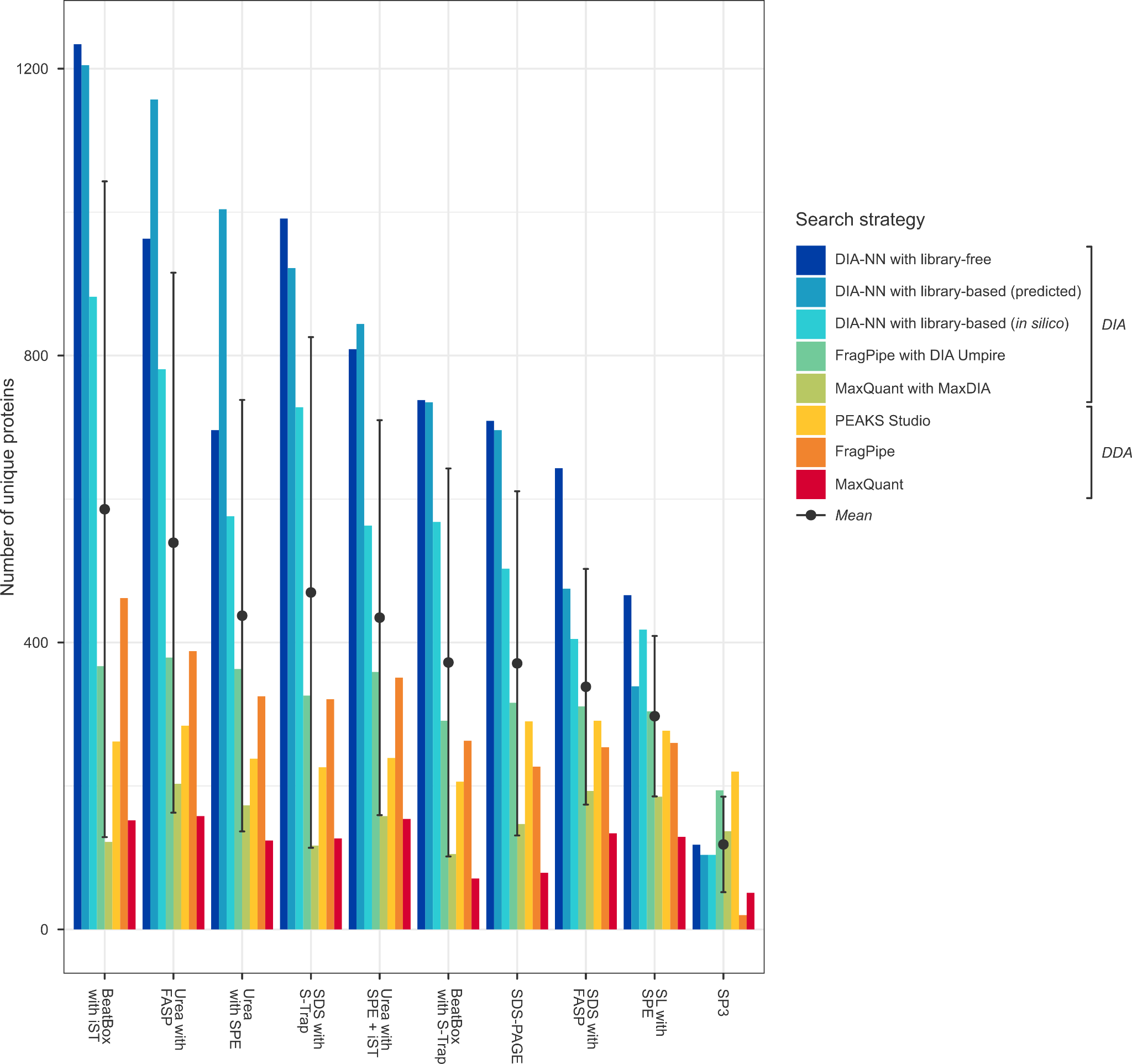
Fig S1. Total numbers of proteins retrieved with each search strategy |** Bar chart of numbers of unique proteins identified at 1% FDR and *q*-value ≤ .01 in the DIA and DDA data, summed per and coloured by search strategy (from left to right, highest to lowest total proteins). Black dots represent mean values per protocol, with error bars reflecting the standard deviation.

**
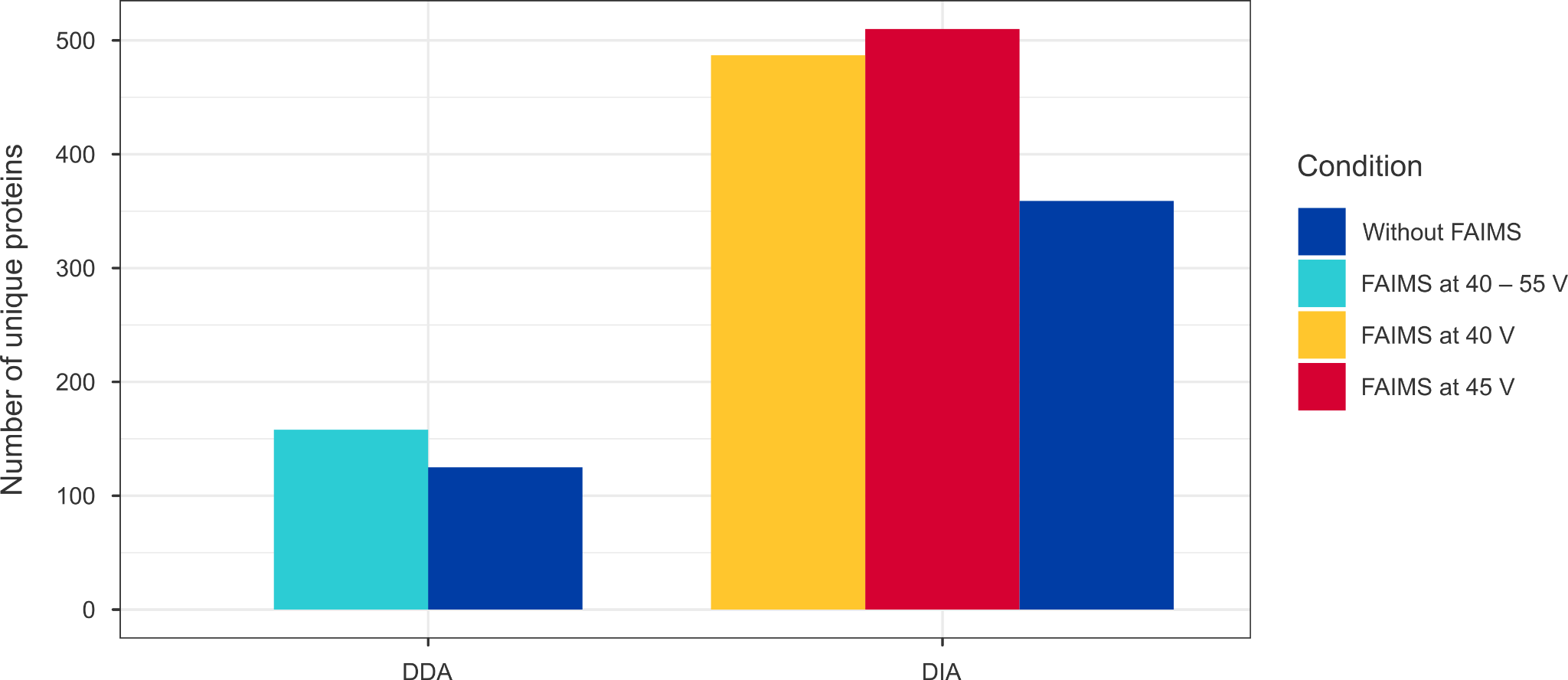
Fig S2. Impact of FAIMS on protein identification |** Bar chart of the numbers of unique proteins identified at 1% FDR and *q*-value ≤ .01 in the pooled samples analysed in DDA and DIA modes, summed per and coloured by the use of a high-field asymmetric-waveform ion mobility spectrometry (FAIMS) source with associated negative compensation voltage.

**Table S2. DIA identification results for each protocol |** Total numbers of proteotypic precursors, peptides and proteins identified per protocol at 1% FDR and *q*-value ≤ .01, with total quantities.

| **Protocol** | **Precursors identified** | **Peptides identified** | **Proteins identified** | **Total quantity** |
| --- | --- | --- | --- | --- |
| BeatBox with iST | 2840 | 2645 | 1205 | 2.88E+09 |
| Urea with FASP | 2679 | 2506 | 1157 | 5.48E+09 |
| Urea with SPE | 2433 | 2259 | 1004 | 1.26E+09 |
| SDS with S-Trap | 2402 | 2274 | 922 | 7.16E+09 |
| Urea with SPE + iST | 2178 | 2042 | 844 | 1.07E+09 |
| BeatBox with S-Trap | 2029 | 1900 | 735 | 1.45E+09 |
| SDS-PAGE | 1953 | 1820 | 696 | 6.94E+08 |
| SDS with FASP | 1500 | 1369 | 475 | 1.97E+09 |
| SL with SPE | 1139 | 1066 | 339 | 1.89E+09 |
| SP3 | 328 | 303 | 104 | 1.68E+08 |

**Table S3. Statistical over-representation of protein classes |** Over-representation test results for proteins recovered by every protocol (*n* = 98). Only statistically significant results (i.e., Bonferroni-corrected for *p* ≤ .05) for classified proteins are shown. Parent classes (*italics*) are indented below subclasses to illustrate hierarchical relationships.

| **Protein Class** | **# Reference genes** | **# Queried genes** | **# Expected**  **genes** | **Fold Enrichment** | ***p*-Value** |
| --- | --- | --- | --- | --- | --- |
| Tubulin | 17 | 3 | 0.11 | 26.68 | 3.35 x 10^-2^ |
| Intermediate filament | 25 | 4 | 0.17 | 24.19 | 3.85 x 10^-3^ |
| Small GTPase | 112 | 9 | 0.74 | 12.15 | 9.82 x 10^-6^ |
| *⤷ G-protein* | 166 | 13 | 1.10 | 11.84 | 1.21 x 10^-8^ |
| *⤷ Protein-binding activity modulator* | 673 | 15 | 4.45 | 3.37 | 6.36 x 10^-3^ |


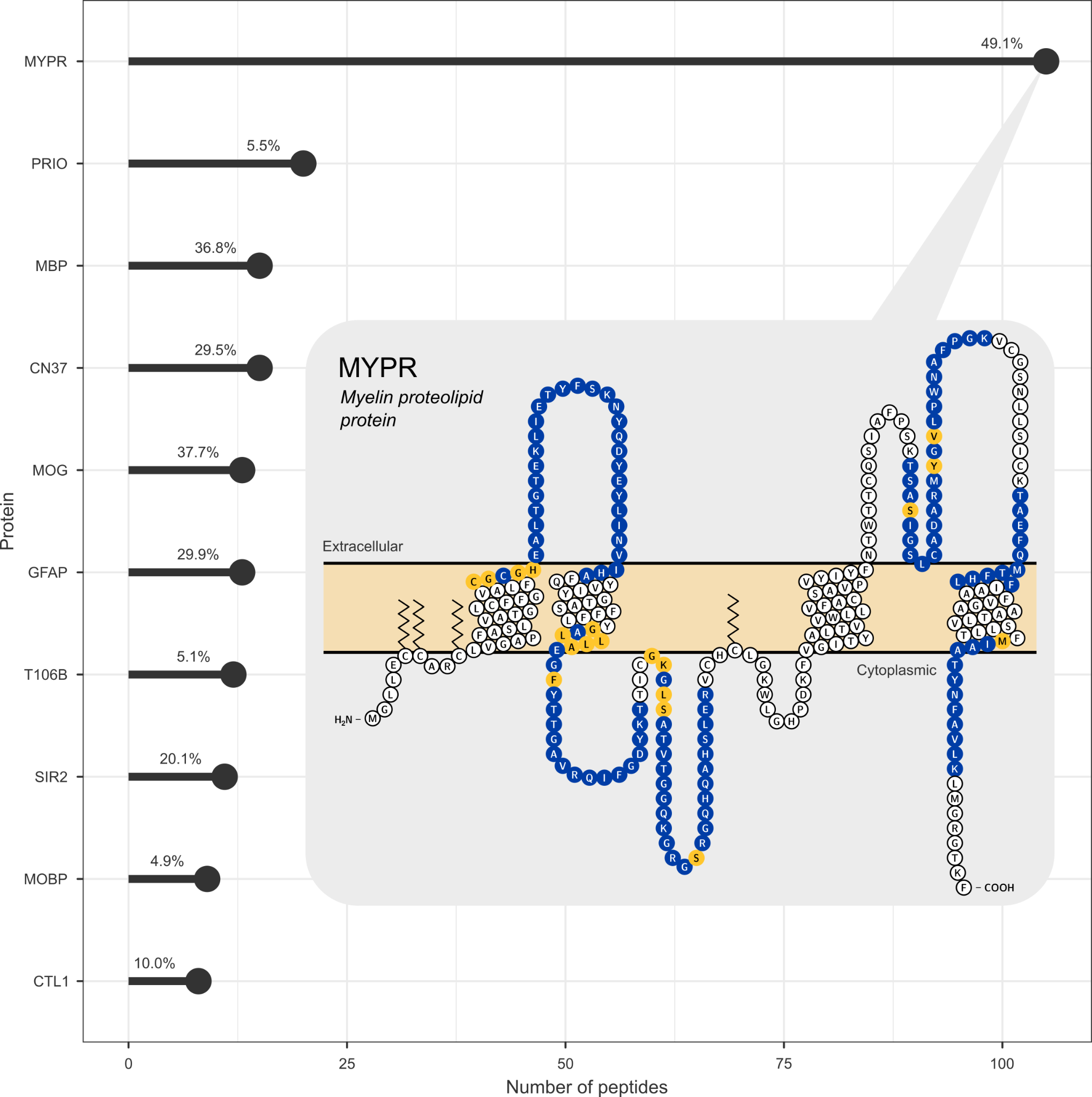
**Fig S3.** **Nature of non-tryptic peptides |** Proteins associated with the ten highest numbers of non-tryptic peptides, and their percentage sequence coverage. Myelin proteolipid protein (MYPR), the most abundant and the major transmembrane protein of the central nervous system, is illustrated by its proteoform, with (top to bottom) the extracellular, transmembrane and cytoplasmic spaces; created with Protter (v.1.0) [[7]](https://paperpile.com/c/6UBX72/fVMoO). Solid colours represent recovered peptides, with yellow residues reflecting non-tryptic cleavage sites.

**Table S4. Nature of dependent peptides |** Total numbers per protocol of annotated modifications to ≥ 2 unique peptides, identified by dependent peptide search. Fixed modifications (i.e., those also explicitly searched for) are italicised.

| **Modification** | **BB w/**  **iST** | **Urea w/**  **FASP** | **Urea w/ SPE** | **SDS w/**  **S-Trap** | **Urea w/ SPE + iST** | **BB w/**  **S-Trap** | **SDS-**  **PAGE** | **SDS w/ FASP** | **SL w/ SPE** | **SP3** | **Total** |
| --- | --- | --- | --- | --- | --- | --- | --- | --- | --- | --- | --- |
| *Deamidation* | *9* | *4* | *7* | *5* | *7* | *2* | *3* | *4* | *9* | *1* | *51* |
| Comp: 12C-13C | 7 | 5 | 4 | 3 | 3 | 1 | 1 | 2 | 2 | 0 | 28 |
| *Oxidation* | *6* | *4* | *1* | *3* | *5* | *0* | *2* | *0* | *1* | *0* | *22* |
| *Carbamido-methylation* | *0* | *0* | *7* | *0* | *10* | *0* | *0* | *1* | *0* | *0* | *18* |
| Loss of water | 6 | 1 | 1 | 1 | 3 | 0 | 0 | 0 | 2 | 0 | 14 |
| Acetaldehyde | 0 | 1 | 5 | 0 | 1 | 0 | 0 | 0 | 4 | 0 | 11 |
| Comp: O-NH_3_ | 4 | 0 | 1 | 1 | 1 | 0 | 0 | 1 | 0 | 0 | 8 |
| Comp: -H_5_C_2_NOS | 1 | 0 | 1 | 1 | 1 | 0 | 0 | 1 | 1 | 1 | 7 |
| Comp: 13C-12C | 1 | 2 | 2 | 0 | 2 | 0 | 0 | 0 | 0 | 0 | 7 |
| Comp: O-H_2_ | 3 | 1 | 0 | 0 | 0 | 0 | 0 | 2 | 1 | 0 | 7 |
| Didehydro | 1 | 1 | 1 | 0 | 1 | 0 | 0 | 2 | 1 | 0 | 7 |
| Loss of ammonia | 2 | 0 | 2 | 0 | 2 | 0 | 0 | 1 | 0 | 0 | 7 |
| Reduction | 0 | 1 | 2 | 0 | 1 | 0 | 0 | 2 | 0 | 0 | 6 |
| Comp: O-CH_2_ | 1 | 0 | 1 | 0 | 0 | 0 | 1 | 0 | 1 | 0 | 4 |
| Comp: S-C_4_ | 3 | 0 | 0 | 0 | 0 | 0 | 1 | 0 | 0 | 0 | 4 |
| Formylation | 1 | 1 | 1 | 0 | 1 | 0 | 0 | 0 | 0 | 0 | 4 |
| Comp:C-N_3_ | 2 | 0 | 0 | 0 | 1 | 0 | 0 | 0 | 0 | 0 | 3 |
| Sulphide | 0 | 0 | 1 | 0 | 0 | 0 | 0 | 1 | 1 | 0 | 3 |
| Comp: C | 0 | 1 | 1 | 0 | 0 | 0 | 0 | 0 | 0 | 0 | 2 |
| Comp: NO-CH_3_ | 0 | 2 | 0 | 0 | 0 | 0 | 0 | 0 | 0 | 0 | 2 |
| Carboxy-  methylation | 0 | 0 | 0 | 0 | 2 | 0 | 0 | 0 | 0 | 0 | 2 |

**Table S5. Summary of hydropathy by protocol |** Summary statistics for GRand AVerage of hydropathY (GRAVY) score for proteins retrieved in all protocols (the “common proteome”, italicised; *n* = 98), and peptides corresponding to the same proteins retrieved by each protocol.

| **Protocol** | **Mean** | **Standard deviation (SD)** | **Median** |
| --- | --- | --- | --- |
| *Common proteome* | *– 0.262* | *0.416* | *– 0.322* |
| BeatBox with iST | – 0.395 | 0.848 | – 0.340 |
| Urea with FASP | – 0.413 | 0.809 | – 0.422 |
| Urea with SPE | – 0.341 | 0.783 | – 0.300 |
| SDS with S-Trap | – 0.326 | 0.852 | – 0.262 |
| Urea with SPE + iST | – 0.318 | 0.799 | – 0.267 |
| BeatBox with S-Trap | – 0.344 | 0.876 | – 0.259 |
| SDS-PAGE | – 0.341 | 0.846 | – 0.300 |
| SDS with FASP | – 0.600 | 0.863 | – 0.668 |
| SL with SPE | – 0.517 | 0.838 | – 0.514 |
| SP3 | – 0.345 | 0.820 | – 0.300 |

**Table S6. Hydropathic profiles retrieved by each protocol |** Results of *post-hoc* pairwise comparisons (*n* = 55) with Bonferroni correction at 95% CI for a linear mixed effects model with Satterthwaite *t*-tests (*n*_obs_ = 4,383) with respect to GRAVY score, for proteins shared across all protocols (“common proteome”, CP) and for peptides corresponding to the same proteins retrieved by each protocol. Cells containing *p*-values are coloured based on the following significance codes: | < .0001 | ≤ .001 | ≤ .01 | ≤ .05 | non-significant |

| **Comparison** | **Estimate (*E*)** | **Standard error (SE)** | ***t*-Ratio** | ***p*-Value** |
| --- | --- | --- | --- | --- |
| CP - BB_iST | 0.147533262 | 0.09068671 | 1.62684550 | 1.00000 |
| CP - UREA_FASP | 0.165035663 | 0.09051606 | 1.82327489 | 1.00000 |
| CP - UREA_SPE | 0.093950266 | 0.09085167 | 1.03410607 | 1.00000 |
| CP - SDS_STRAP | 0.077187294 | 0.09213778 | 0.83773776 | 1.00000 |
| CP - UREA_SPE+iST | 0.070473672 | 0.09169707 | 0.76854875 | 1.00000 |
| CP - BB_STRAP | 0.095514783 | 0.09275937 | 1.02970500 | 1.00000 |
| CP - SDS-PAGE | 0.092605962 | 0.09185701 | 1.00815342 | 1.00000 |
| CP - SDS_FASP | 0.351403661 | 0.09247945 | 3.79980265 | 0.00808 |
| CP - SL | 0.266768444 | 0.09345075 | 2.85464220 | 0.23812 |
| CP - SP3 | 0.088019655 | 0.10115035 | 0.87018638 | 1.00000 |
| BB_iST - UREA_FASP | 0.017502401 | 0.05047937 | 0.34672381 | 1.00000 |
| BB_iST - UREA_SPE | – 0.053582995 | 0.05108762 | – 1.04884503 | 1.00000 |
| BB_iST - SDS_STRAP | – 0.070345967 | 0.05340744 | – 1.31715671 | 1.00000 |
| BB_iST - UREA_SPE+iST | – 0.077059589 | 0.05262056 | – 1.46443870 | 1.00000 |
| BB_iST - BB_STRAP | – 0.052018479 | 0.05450380 | – 0.95440088 | 1.00000 |
| BB_iST - INGEL | – 0.054927300 | 0.05290709 | – 1.03818413 | 1.00000 |
| BB_iST - SDS_FASP | 0.203870399 | 0.05401199 | 3.77453978 | 0.00894 |
| BB_iST - SL | 0.119235183 | 0.05570590 | 2.14044070 | 1.00000 |
| BB_iST - SP3 | – 0.059513606 | 0.06815198 | – 0.87324842 | 1.00000 |
| UREA_FASP - UREA_SPE | – 0.071085396 | 0.05078409 | – 1.39975734 | 1.00000 |
| UREA_FASP - SDS_STRAP | – 0.087848368 | 0.05311716 | – 1.65386029 | 1.00000 |
| UREA_FASP - UREA_SPE+iST | – 0.094561990 | 0.05232592 | – 1.80717290 | 1.00000 |
| UREA_FASP - BB_STRAP | – 0.069520880 | 0.05421940 | – 1.28221414 | 1.00000 |
| UREA_FASP - SDS-PAGE | – 0.072429701 | 0.05261405 | – 1.37662273 | 1.00000 |
| UREA_FASP - SDS_FASP | 0.186367998 | 0.05372498 | 3.46892635 | 0.02904 |
| UREA_FASP - SL | 0.101732782 | 0.05542767 | 1.83541521 | 1.00000 |
| UREA_FASP - SP3 | – 0.077016007 | 0.06792474 | – 1.13384322 | 1.00000 |
| UREA_SPE - SDS_STRAP | – 0.016762972 | 0.05368708 | – 0.31223477 | 1.00000 |
| UREA_SPE - UREA_SPE+iST | – 0.023476594 | 0.05290436 | – 0.44375538 | 1.00000 |
| UREA_SPE - BB_STRAP | 0.001564516 | 0.05477784 | 0.02856112 | 1.00000 |
| UREA_SPE - SDS-PAGE | – 0.001344304 | 0.05318936 | – 0.02527394 | 1.00000 |
| UREA_SPE - SDS_FASP | 0.257453394 | 0.05428851 | 4.74231819 | 0.00012 |
| UREA_SPE - SL | 0.172818178 | 0.05597406 | 3.08746915 | 0.11179 |
| UREA_SPE - SP3 | – 0.005930611 | 0.06837134 | – 0.08674119 | 1.00000 |
| SDS_STRAP - UREA_SPE+iST | – 0.006713622 | 0.05510562 | – 0.12183190 | 1.00000 |
| SDS_STRAP - BB_STRAP | 0.018327488 | 0.05688546 | 0.32218230 | 1.00000 |
| SDS_STRAP - SDS-PAGE | 0.015418668 | 0.05537135 | 0.27845930 | 1.00000 |
| SDS_STRAP - SDS_FASP | 0.274216366 | 0.05641441 | 4.86075030 | 0.00007 |
| SDS_STRAP - SL | 0.189581150 | 0.05803825 | 3.26648628 | 0.06040 |
| SDS_STRAP - SP3 | 0.010832361 | 0.07007127 | 0.15459062 | 1.00000 |
| UREA_SPE+iST - BB_STRAP | 0.025041111 | 0.05616885 | 0.44581850 | 1.00000 |
| UREA_SPE+iST - SDS-PAGE | 0.022132290 | 0.05462083 | 0.40519874 | 1.00000 |
| UREA_SPE+iST - SDS_FASP | 0.280929989 | 0.05569174 | 5.04437478 | 0.00003 |
| UREA_SPE+iST - SL | 0.196294772 | 0.05733605 | 3.42358402 | 0.03433 |
| UREA_SPE+iST - SP3 | 0.017545983 | 0.06949077 | 0.25249374 | 1.00000 |
| BB_STRAP - SDS-PAGE | – 0.002908821 | 0.05642957 | – 0.05154781 | 1.00000 |
| BB_STRAP - SDS_FASP | 0.255888878 | 0.05743722 | 4.45510538 | 0.00047 |
| BB_STRAP - SL | 0.171253662 | 0.05902007 | 2.90161735 | 0.20532 |
| BB_STRAP - SP3 | – 0.007495127 | 0.07088662 | – 0.10573402 | 1.00000 |
| SDS-PAGE - SDS_FASP | 0.258797699 | 0.05595469 | 4.62513007 | 0.00021 |
| SDS-PAGE - SL | 0.174162482 | 0.05759149 | 3.02410105 | 0.13803 |
| SDS-PAGE - SP3 | – 0.004586306 | 0.06970168 | – 0.06579908 | 1.00000 |
| SDS_FASP - SL | – 0.084635216 | 0.05857916 | – 1.44480089 | 1.00000 |
| SDS_FASP - SP3 | – 0.263384005 | 0.07051994 | – 3.73488705 | 0.01047 |
| SL - SP3 | – 0.178748789 | 0.07178896 | – 2.48992025 | 0.70491 |

**Table S7. Summary of isoelectric point by protocol |** Summary statistics for pI for proteins retrieved in all protocols (the “common proteome”, italicised; *n* = 98), and peptides corresponding to the same proteins retrieved by each protocol.

| **Protocol** | **Mean** | **Standard deviation (SD)** | **Median** |
| --- | --- | --- | --- |
| *Common proteome* | *6.596* | *1.770* | *6.086* |
| BeatBox with iST | 5.928 | 1.943 | 6.067 |
| Urea with FASP | 5.983 | 1.997 | 5.993 |
| Urea with SPE | 6.045 | 2.029 | 6.089 |
| SDS with S-Trap | 6.233 | 2.053 | 6.213 |
| Urea with SPE + iST | 5.808 | 1.850 | 5.461 |
| BeatBox with S-Trap | 6.339 | 2.081 | 6.327 |
| SDS-PAGE | 6.296 | 2.039 | 6.234 |
| SDS with FASP | 5.860 | 1.952 | 5.410 |
| SL with SPE | 6.039 | 2.074 | 5.910 |
| SP3 | 6.029 | 1.955 | 6.108 |

**Table S8. Isoelectric profiles retrieved by each protocol |** Results of *post-hoc* pairwise comparisons (*n* = 55) with Bonferroni correction at 95% CI for a linear mixed effects model with Satterthwaite *t*-tests (*n*_obs_ = 4,383) with respect to average pI, for proteins shared across all protocols (“common proteome”, CP) and for peptides corresponding to the same proteins retrieved by each protocol. Cells containing *p*-values are coloured based on the following significance codes: | < .0001 | ≤ .001 | ≤ .01 | ≤ .05 | non-significant |

| **Comparison** | **Estimate (*E*)** | **Standard error (SE)** | ***t*-Ratio** | ***p*-Value** |
| --- | --- | --- | --- | --- |
| CP - BB_iST | 0.875861523 | 0.2164021 | 4.04737981 | 0.00290 |
| CP - UREA_FASP | 0.820913996 | 0.2160157 | 3.80025138 | 0.00806 |
| CP - UREA_SPE | 0.760144403 | 0.2167766 | 3.50658035 | 0.02523 |
| CP - SDS_STRAP | 0.567539927 | 0.2197062 | 2.58317635 | 0.54028 |
| CP - UREA_SPE+iST | 0.995618784 | 0.2187001 | 4.55243903 | 0.00030 |
| CP - BB_STRAP | 0.459513188 | 0.2211299 | 2.07802365 | 1.00000 |
| CP - SDS-PAGE | 0.504676063 | 0.2190648 | 2.30377536 | 1.00000 |
| CP - SDS_FASP | 0.939760786 | 0.2204882 | 4.26218149 | 0.00114 |
| CP - SL | 0.755258829 | 0.2227174 | 3.39110827 | 0.03864 |
| CP - SP3 | 0.700195798 | 0.2404456 | 2.91207544 | 0.19855 |
| BB_iST - UREA_FASP | – 0.054947527 | 0.1195788 | – 0.45950897 | 1.00000 |
| BB_iST - UREA_SPE | – 0.115717120 | 0.1210166 | – 0.95620855 | 1.00000 |
| BB_iST - SDS_STRAP | – 0.308321595 | 0.1266728 | – 2.43400000 | 0.82373 |
| BB_iST - UREA_SPE+iST | 0.119757261 | 0.1247586 | 0.95991182 | 1.00000 |
| BB_iST - BB_STRAP | – 0.416348335 | 0.1293315 | – 3.21923383 | 0.07126 |
| BB_iST - INGEL | – 0.371185460 | 0.1254564 | – 2.95868021 | 0.17094 |
| BB_iST - SDS_FASP | 0.063899263 | 0.1281399 | 0.49866796 | 1.00000 |
| BB_iST - SL | – 0.120602693 | 0.1322394 | – 0.91200296 | 1.00000 |
| BB_iST - SP3 | – 0.175665725 | 0.1622561 | – 1.08264482 | 1.00000 |
| UREA_FASP - UREA_SPE | – 0.060769593 | 0.1203243 | – 0.50504842 | 1.00000 |
| UREA_FASP - SDS_STRAP | – 0.253374069 | 0.1260116 | – 2.01072089 | 1.00000 |
| UREA_FASP - UREA_SPE+iST | 0.174704788 | 0.1240872 | 1.40791991 | 1.00000 |
| UREA_FASP - BB_STRAP | – 0.361400808 | 0.1286839 | – 2.80843804 | 0.27513 |
| UREA_FASP - SDS-PAGE | – 0.316237933 | 0.1247887 | – 2.53418640 | 0.62200 |
| UREA_FASP - SDS_FASP | 0.118846790 | 0.1274863 | 0.93223209 | 1.00000 |
| UREA_FASP - SL | – 0.065655167 | 0.1316061 | – 0.49887634 | 1.00000 |
| UREA_FASP - SP3 | – 0.120718198 | 0.1617404 | – 0.74637011 | 1.00000 |
| UREA_SPE - SDS_STRAP | – 0.192604476 | 0.1273114 | – 1.51286084 | 1.00000 |
| UREA_SPE - UREA_SPE+iST | 0.235474381 | 0.1254070 | 1.87768155 | 1.00000 |
| UREA_SPE - BB_STRAP | – 0.300631216 | 0.1299571 | – 2.31331186 | 1.00000 |
| UREA_SPE - SDS-PAGE | – 0.255468340 | 0.1261012 | – 2.02589902 | 1.00000 |
| UREA_SPE - SDS_FASP | 0.179616383 | 0.1287713 | 1.39484842 | 1.00000 |
| UREA_SPE - SL | – 0.004885574 | 0.1328512 | – 0.03677477 | 1.00000 |
| UREA_SPE - SP3 | – 0.059948605 | 0.1627552 | – 0.36833612 | 1.00000 |
| SDS_STRAP - UREA_SPE+iST | 0.428078857 | 0.1305598 | 3.27879627 | 0.05784 |
| SDS_STRAP - BB_STRAP | – 0.108026740 | 0.1347872 | – 0.80146140 | 1.00000 |
| SDS_STRAP - SDS-PAGE | – 0.062863864 | 0.1311698 | – 0.47925575 | 1.00000 |
| SDS_STRAP - SDS_FASP | 0.372220859 | 0.1336443 | 2.78516162 | 0.29568 |
| SDS_STRAP - SL | 0.187718902 | 0.1375798 | 1.36443644 | 1.00000 |
| SDS_STRAP - SP3 | 0.132655870 | 0.1666373 | 0.79607541 | 1.00000 |
| UREA_SPE+iST - BB_STRAP | – 0.536105596 | 0.1331409 | – 4.02660449 | 0.00317 |
| UREA_SPE+iST - SDS-PAGE | – 0.490942721 | 0.1293799 | – 3.79458144 | 0.00826 |
| UREA_SPE+iST - SDS_FASP | – 0.055857998 | 0.1319837 | – 0.42321903 | 1.00000 |
| UREA_SPE+iST - SL | – 0.240359955 | 0.1359673 | – 1.76777783 | 1.00000 |
| UREA_SPE+iST - SP3 | – 0.295422986 | 0.1653085 | – 1.78710118 | 1.00000 |
| BB_STRAP - SDS-PAGE | 0.045162875 | 0.1337391 | 0.33769389 | 1.00000 |
| BB_STRAP - SDS_FASP | 0.480247598 | 0.1360581 | 3.52972344 | 0.02314 |
| BB_STRAP - SL | 0.295745642 | 0.1398421 | 2.11485396 | 1.00000 |
| BB_STRAP - SP3 | 0.240682610 | 0.1685100 | 1.42829899 | 1.00000 |
| SDS-PAGE - SDS_FASP | 0.435084723 | 0.1325871 | 3.28150072 | 0.05729 |
| SDS-PAGE - SL | 0.250582766 | 0.1365531 | 1.83505682 | 1.00000 |
| SDS-PAGE - SP3 | 0.195519735 | 0.1657907 | 1.17931677 | 1.00000 |
| SDS_FASP - SL | – 0.184501956 | 0.1388252 | – 1.32902372 | 1.00000 |
| SDS_FASP - SP3 | – 0.239564988 | 0.1676670 | – 1.42881420 | 1.00000 |
| SL - SP3 | – 0.055063032 | 0.1705878 | – 0.32278403 | 1.00000 |

# References

1. [Demichev V, Messner CB, Vernardis SI, Lilley KS, Ralser M. DIA-NN: neural networks and interference correction enable deep proteome coverage in high throughput. Nat Methods. 2020;17: 41–44.](http://paperpile.com/b/6UBX72/ASRA)

2. [Yu F, Teo GC, Kong AT, Fröhlich K, Li GX, Demichev V, et al. Analysis of DIA proteomics data using MSFragger-DIA and FragPipe computational platform. Nat Commun. 2023;14: 4154.](http://paperpile.com/b/6UBX72/Aoxl)

3. [Tsou C-C, Avtonomov D, Larsen B, Tucholska M, Choi H, Gingras A-C, et al. DIA-Umpire: comprehensive computational framework for data-independent acquisition proteomics. Nat Methods. 2015;12: 258–64, 7 p following 264.](http://paperpile.com/b/6UBX72/dH3I)

4. [Tyanova S, Temu T, Cox J. The MaxQuant computational platform for mass spectrometry-based shotgun proteomics. Nat Protoc. 2016;11: 2301–2319.](http://paperpile.com/b/6UBX72/Os8R)

5. [Sinitcyn P, Hamzeiy H, Salinas Soto F, Itzhak D, McCarthy F, Wichmann C, et al. MaxDIA enables library-based and library-free data-independent acquisition proteomics. Nat Biotechnol. 2021;39: 1563–1573.](http://paperpile.com/b/6UBX72/KL33)

6. [Yu F, Haynes SE, Nesvizhskii AI. IonQuant enables accurate and sensitive label-free quantification with FDR-controlled match-between-runs. Mol Cell Proteomics. 2021;20: 100077.](http://paperpile.com/b/6UBX72/bSzI)

7. [Omasits U, Ahrens CH, Müller S, Wollscheid B. Protter: interactive protein feature visualization and integration with experimental proteomic data. Bioinformatics. 2014;30: 884–886.](http://paperpile.com/b/6UBX72/fVMoO)
